# Galectin-8 induces functional disease markers in human osteoarthritis and cooperates with galectins-1 and -3

**DOI:** 10.1007/s00018-018-2856-2

**Published:** 2018-06-22

**Authors:** Daniela Weinmann, Michael Kenn, Sebastian Schmidt, Katy Schmidt, Sonja M. Walzer, Bernd Kubista, Reinhard Windhager, Wolfgang Schreiner, Stefan Toegel, Hans-Joachim Gabius

**Affiliations:** 1 Karl Chiari Lab for Orthopaedic Biology, Department of Orthopedics and Trauma Surgery, Medical University of Vienna, Waehringer Guertel 18-20, 1090 Vienna, Austria; 2 Center for Medical Statistics, Informatics and Intelligent Systems, Institute of Biosimulation and Bioinformatics, Medical University of Vienna, Vienna, Austria; 3 Institute of Physiological Chemistry, Faculty of Veterinary Medicine, Ludwig-Maximilians-University Munich, Munich, Germany; 4 Center for Anatomy and Cell Biology, Department for Cell and Developmental Biology, Medical University of Vienna, Vienna, Austria; 5 Ludwig Boltzmann Cluster for Arthritis and Rehabilitation, Vienna, Austria

**Keywords:** Chondrocytes, Lectin, Inflammation, Interleukin, NF-κB

## Abstract

The reading of glycan-encoded signals by tissue lectins is considered a major route of the flow of biological information in many (patho)physiological processes. The arising challenge for current research is to proceed from work on a distinct protein to family-wide testing of lectin function. Having previously identified homodimeric galectin-1 and chimera-type galectin-3 as molecular switches in osteoarthritis progression, we here provide proof-of-principle evidence for an intra-network cooperation of galectins with three types of modular architecture. We show that the presence of tandem-repeat-type galectin-8 significantly correlated with cartilage degeneration and that it is secreted by osteoarthritic chondrocytes. Glycan-inhibitable surface binding of galectin-8 to these cells increased gene transcription and the secretion of functional disease markers. The natural variant galectin-8 (F19Y) was less active than the prevalent form. Genome-wide array analysis revealed induction of a pro-degradative/inflammatory gene signature, largely under control of NF-κB signaling. This signature overlapped with respective gene-expression patterns elicited by galectins-1 and -3, but also presented supplementary features. Functional assays with mixtures of galectins that mimic the pathophysiological status unveiled cooperation between the three galectins. Our findings shape the novel concept to consider individual galectins as part of a so far not realized teamwork in osteoarthritis pathogenesis, with relevance beyond this disease.

**Electronic supplementary material:**

The online version of this article (10.1007/s00018-018-2856-2) contains supplementary material, which is available to authorized users.

## Introduction

Cell surfaces are characterized by their glycosylation profile like by a molecular fingerprint. The heightened awareness that the constituents of cellular glycoconjugates convey molecular ‘messages’ relevant for diverse aspects of cell (patho)physiology directs attention to the ‘readers’ of the sugar-encoded signals (i.e., lectins) [1–9]. Indeed, the functional pairing between distinct glycan determinants and their cognate lectins can elicit a broad range of intracellular signaling pathways [10], among them responses typically encountered in osteoarthritis (OA). For instance, chondrocyte apoptosis, impaired cell adhesion or a switch to a pro-degradative/inflammatory microenvironment have been shown to contribute to the clinical manifestation of this most prevalent form of arthritis [11, 12]. In the quest to define the molecular switches responsible for initiating the harmful processes that cause serious discomfort and pain, measuring (i) a shift of the sialylation profile of *N*-glycans in chondrocytes exposed to pro-inflammatory cytokines [13, 14] and (ii) increased tissue reactivity for β-galactosides in regions of severe degeneration [15] has given reason to hypothesize that respective receptors, i.e. adhesion/growth-regulatory galectins (Gals), are involved in OA pathophysiology.

As a first step to test this hypothesis, we have recently provided evidence for expression of galectins in human OA chondrocytes in vitro and in situ as well as suggested a correlation of immunohistochemical cell-positivity in clinical specimens with the degree of degeneration defined by the Mankin score (MS) [16]. Intriguingly, chondrocytes in non-OA diseases considered as controls were immunonegative [16]. Equally significant, Gal-1 [17] and Gal-3 [18] were both revealed to enhance the availability of functional disease markers. These data not only identified an active role of two galectins in driving OA pathogenesis but also provided initial insights into the possibility of a concerted action of galectins, a concept of significance beyond this disease. After all, galectins have been delineated to be broadly active in immune regulation in an apparent context-dependent manner, but have mostly been studied separately until now [19–23]. Since we have previously detected Gal-8 in sections of OA cartilage in a systematic galectin profiling [16], answering the question about its pathophysiological role in this disease will put the concept of a functional teamwork within the galectin family to the test in a proof-of-principle manner.

Human Gal-8 is a tandem-repeat type family member with two different carbohydrate recognition domains (CRDs) connected by a linker peptide of either 33 amino acids (Gal-8S) or 75 amino acids (Gal-8L) [24–26]. It is abundantly expressed in human tissues [27, 28] and an integral part of the galectin signature of many tumor types such as colon, head and neck, prostate and urothelial carcinomas [29–34]. Cell biologically, it acts as matricellular protein: it can reduce cell adhesion when present in excess in solution by blocking access to integrins or promote cell attachment when adsorbed to a substratum [35]. Due to its functional bivalency it may serve as *trans*-bridging molecule in solution [36–38] or as *cis*-crosslinker at the cell surface.

The hereby resulting response profile of cells involved in immunity is complex and cannot be simply extrapolated from one cell type to the other. On the one hand, Gal-8 has a pro-apoptotic effect on immature CD4^high^CD8^high^ thymocytes [39] and causes phosphatidylserine exposure on activated Th17 and promyelocytic tumor (HL60) cells as well as synovial fluid cells of rheumatoid arthritis patients [40–42]. In addition, its absence in KO mice is associated with earlier on-set and more severe manifestation of experimental autoimmune encephalomyelitis, highly significantly raising the abundance of CXCR3^+^ (Th1-like) regulatory T cells in these animals [42]. On the other hand, in contrast to these indications for immunosuppressive activity, Gal-8 is strongly co-stimulatory (at low concentration; 0.1 µM and below) or proliferative (at high concentration; 0.5–2 µM) in murine naïve T cells, enhancing IL-2, IL-4 and IFN-γ gene transcription [43, 44]. Potent activation of neutrophils with evidence for accelerated MMP-3-mediated proMMP-9 processing [45], of platelets [46] and of microvascular endothelial cells [47] attests a Janus-like immunostimulatory capacity. In the case of B cells, Gal-8 positively affects plasma cell formation and antibody production [48]. Beyond immune cells, the versatility of Gal-8 is underscored by recent findings of (i) osteoclastogenic efficiency through increasing RANKL availability, when adding Gal-8 to co-cultures of murine osteoblasts and bone marrow cells, flanked by in vivo work using overexpressing transgenic and knock-out mice [49, 50] and (ii) its involvement in early stages of avian limb morphogenesis where chicken Gal-8 was first detectable in condensing precartilage mesenchyme, later present in the osteoprogenitor layer and eventually in osteoblasts [51, 52].

This wealth of reports on Gal-8 as multifunctional effector prompts a detailed investigation of (i) the putative correlation between chondrocyte immunopositivity for Gal-8 and cartilage degeneration [16], (ii) the possibility of a role of Gal-8 in exacerbating OA-associated disease parameters, and, should this be the case, (iii) an interplay of Gal-8 with Gal-1 and/or -3. Thus, in this study, we pursued the following stepwise strategy to address these issues.

First, we validated the correlation of increasing immunopositivity for Gal-8 with disease progression in clinical cartilage specimens. We then examined the carbohydrate-inhibitable Gal-8 binding to OA chondrocytes and its effect on functional disease markers including an analysis of the impact of linker length and linker cleavage as well as of presence of the F19Y substitution due to a single nucleotide polymorphism (SNP). This seemingly subtle change is associated with rheumatoid arthritis with antagonistic pleiotropy [53]. Transcriptomics, bioinformatics, and NF-κB-targeted blocking studies followed to characterize Gal-8-induced alterations of gene expression in OA chondrocytes and underlying mechanistic details. The emerging results intimated the possibility for a functional cooperation with Gal-1 and -3 in situ that we finally tested experimentally by co-treatment of OA chondrocytes with galectin mixtures.

## Results

### Gal-8 presence correlates with disease progression

**Fig. 1:**
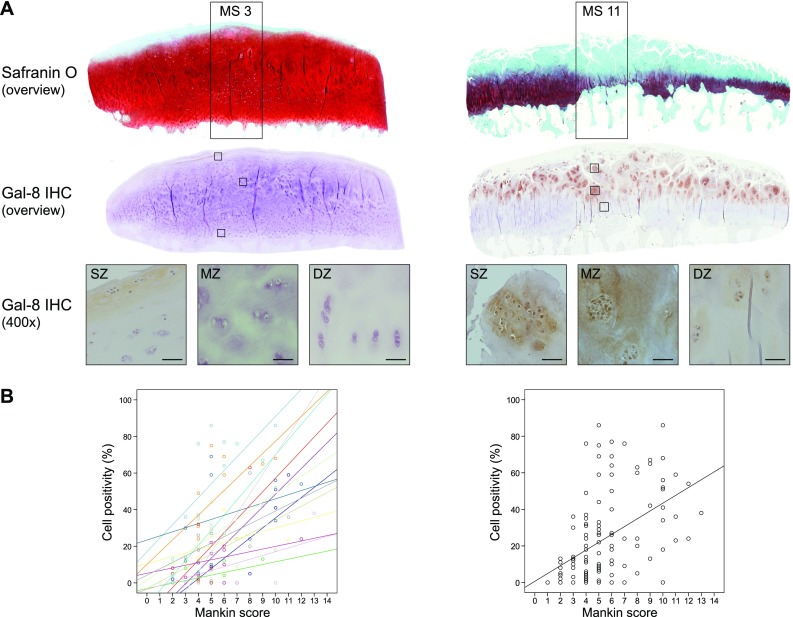
Presence of Gal-8 in OA chondrocytes correlates with the degeneration status of articular cartilage. Femoral condyles were obtained from 15 OA patients who underwent arthroplasty. **a** Histological sections were stained with Safranin O. From each patient, five to nine regions of interest were graded according to the Mankin score (MS). Shown are two representative tissue sections of specimen with MS 3 and MS 11, respectively. Consecutive counterstained sections were immunohistochemically processed for Gal-8 detection (brown). Overview images were photomerged using Photoshop from single microphotographs recorded at ×40 magnification. In addition, examples of Gal-8 staining in the superficial, middle and deep zones (SZ, MZ, DZ) of cartilage are shown at ×400 magnification. Scale bars: 50 µm. **b** Shown are scatterplots of MS versus percentage of Gal-8-positive chondrocytes with regression lines for each patient (left panel) and averaged over all patients (right panel). Individual Pearson’s correlation coefficients ranged from 0.35 to 0.96 (mean 0.62 ± 0.16)

Having previously shown a tendency for a higher percentage of Gal-8-positive chondrocytes in MS ≥ 9 than MS ≤ 4 regions and having also reported absence of immunopositivity in specimens of articular cartilage from four osteosarcoma patients, we extended this immunohistochemical analysis by higher patient numbers (15 donors), by including a wide range of degree of cartilage degeneration (from MS 1 to MS 13) and by applying a more detailed approach considering the exact MS of all histological sections analyzed. Exemplary illustrations of Safranin O staining of regions with MS 3 and MS 11 as well as immunopositivity for Gal-8 of chondrocytes from three sites (superficial, middle and deep zones) at two levels of magnification are presented in Fig. 1a (illustration of control to exclude generation of antigen-independent staining given in Supplementary File 1). Whereas chondrons in the superficial zone of OA cartilage (whenever present) were positive for Gal-8 in all specimens analyzed, chondrocytes in the middle and deep zones were immunopositive particularly in regions of severe degradation (Fig. 1a). When plotting the complete set of immunopositivities within the chondrocyte population to the corresponding MS, a positive correlation was delineated, both for each patient (left part) and for the entire group (right part) (*p* < 0.001, Wilcoxon test; Fig. 1b).

At this stage of the investigation, the relative abundance of the two Gal-8 isoforms, distinguished by the linker length, needed to be addressed, because both proteins, i.e., Gal-8S and Gal-8L, would be immunoreactive to the applied antibody. The scheme of the exon structure in the mRNAs of these two versions (Supplementary File 2A) explains the origin of the difference in linker length by alternative splicing. Using three pairs of primers that exploit the presence of the additional exon in the long form (Gal-8L) (Supplementary File 2A), the lengths of the obtained RT-qPCR products invariably corresponded to the standard (or short) type of mRNA that codes for Gal-8S (Supplementary File 2B). On the protein level, Western blot (WB) analysis calibrated with marker and both recombinant Gal-8 proteins yielded a strong signal for Gal-8S from OA chondrocyte extracts (Supplementary File 2C). A very weak band was present at about 40 kDa, close to the position of Gal-8L, and an additional signal was obtained at about the position of a Gal-8S dimer (Supplementary File 2C). The presented results of RT-qPCR and WB analyses identified Gal-8S as the major cellular form of this galectin in OA chondrocytes. Thus, ensuing binding and functional assays were primarily performed with this protein.

### Gal-8 localization in OA chondrons/chondrocytes and its binding

Using laser scanning microscopy and our own anti-Gal-8 antibody preparation, Gal-8 was found to be present throughout the cytoplasm of chondrocytes (Supplementary File 3A). In agreement with the immunohistochemical analysis, Gal-8-positive chondrocytes were localized in cartilage regions with histological signs of osteoarthritic degradation including clefts and depletion of glycosaminoglycans. To become an auto- and/or paracrine effector via cell surface binding, as observed for Gal-1 and -3 [17, 18], two prerequisites must be fulfilled: (i) secretion of the lectin and (ii) its specific binding to the cell surface. Using ELISA, secretion of Gal-8 into the supernatant of isolated OA chondrocytes was detected at a concentration of 1.2 ± 0.3 pg/ml (*n* = 4 patients). To test whether Gal-8 secretion was responsive to a pro-inflammatory stimulus, further experiments were performed in the presence of IL-1β. Of note, addition of IL-1β to the medium did not significantly alter the extent of secretion of Gal-8 (1.4 ± 2.9 pg/ml, *n* = 4 patients). Furthermore, LGALS8 mRNA levels were not altered by IL-1β, TNF-α, or IL-8, as determined using RT-qPCR (data not shown).

Next, binding of the lectin to cell surfaces was examined using a fluorescent derivative of this protein that had been ascertained to maintain carbohydrate-inhibitable binding. When applying labeled galectin as probe to histological cartilage sections, signals were recorded particularly in chondrons located in regions of OA degeneration, with marked tendency to stain cell membranes and the pericellular matrix (Supplementary File 3B). The question of binding of Gal-8 in the presence of Gal-1 or -3 was answered by studying the two-color fluorescence profiles after exposing sections to binary mixtures of labeled galectins. The staining profiles and their overlays that illustrate co-localization at this level of microscopical resolution indicated that OA chondrocytes harbor binding sites for all three tested galectins (Supplementary File 3C–E). Since working at a subsaturating concentration (0.5 µg/ml) with a lectin for which high- and low-affinity binding partners are known and encountering inter-individual variability in primary cultures, competition assays with label-free protein yielded varying extent of blocking galectin binding (data not shown). Considering the obvious positivity and the expectation that the same situation will be observed in cultured chondrocytes, functional assays would be feasible.

**Fig. 2:**
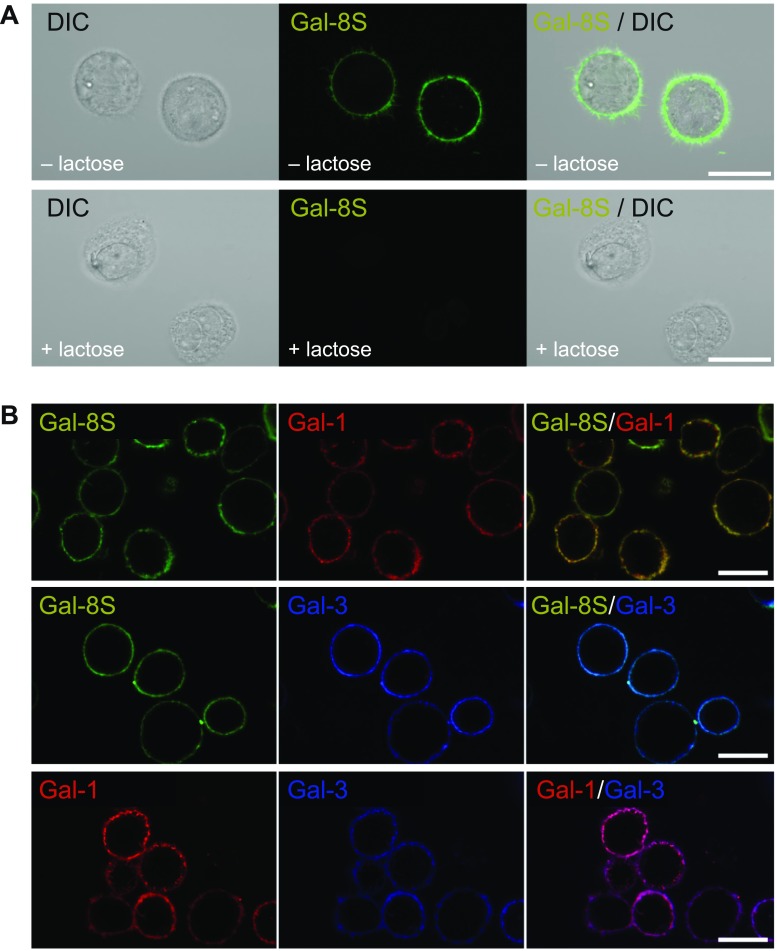
Gal-8S binding sites in isolated OA chondrocytes in vitro. **a** Cultured OA chondrocytes were trypsinized and resuspended prior to labeling with Gal-8S-AlexaFluor488 (green) at 4 °C for 10 min in presence or absence of 0.1 M lactose. After 10 min of incubation, cells were washed and analyzed using laser scanning microscopy, with the focus plane set to the center of cells. Shown are the results from chondrocytes of one patient, representative for experiments with material obtained from three donors. Scale bars: 20 μm. **b** Cultured OA chondrocytes were trypsinized and resuspended prior to labeling with equimolar concentrations of Gal-8S-AlexaFluor488 (green) and Gal-1-AlexaFluor555 (red) (first row), Gal-8S-AlexaFluor488 (green) and Gal-3-AlexaFluor555 (blue) (second row), or Gal-1-AlexaFluor488 (red) and Gal-3-AlexaFluor555 (blue) (third row) at 4 °C. After 10 min of incubation, cells were washed and analyzed using laser scanning microscopy, with the focus plane set to the center of cells. Shown are the results from chondrocytes of one patient, representative for analysis in three independent experimental series (*n* = 3 patients). Scale bars: 20 μm

Testing labeled Gal-8 therefore on isolated viable OA chondrocytes led to strong staining of the membrane (Fig. 2a, upper panel). Of note, signal generation was precluded by addition of the cognate sugar lactose that will occupy the lectin site (Fig. 2a, lower panel). A comparison of Z stack projections of confocal images showed that binding of Gal-8 was confined to the cell surface at 4 °C (Supplementary File 4). At 37 °C (when energy-consuming transport processes can occur), internalization of Gal-8 resulting in a distinct dot-like accumulation of fluorescence intensity within the cells was observed (Supplementary File 5). This capacity of chondrocytes to capture and internalize extracellular/secreted Gal-8 may have a bearing on the level of Gal-8 found in chondrocyte supernatants described above. Binding characteristics of Gal-8 are evocative of the previously reported capacity of Gal-1 and Gal-3 to bind to OA chondrocytes [17, 18]. Figure 2b shows the staining profiles of labeled Gal-1, -3, and -8 at cell surfaces, indicating that isolated chondrocytes present binding sites for these three galectins, in culture and in situ.

Since responses of cells to galectin treatment include induction of apoptosis and massive cytoskeleton rearrangements [54, 55], we examined the cell morphology at this stage microscopically at two levels of magnification. The lactose-inhibitable binding to the cell surface did not trigger any changes when OA chondrocytes were inspected on the ultrastructural level. The overall appearance of treated cultures was indistinguishable from that of control cultures in all cases (Supplementary File 6A). Moreover, transmission electron microscopy did not reveal any noticeable differences between treated and untreated cells (Supplementary File 6B). In particular, the Golgi complex and the ER, important parts of retrograde trafficking after endocytosis, revealed no indication of an alteration. Whether Gal-8 is capable to contribute to disease progression, as intimated by the immunohistochemical observations given in Fig. 1, was studied next by function-oriented in vitro analysis of cells in the presence or absence of Gal-8S.

### Gal-8S binding increases expression of functional disease markers

**Fig. 3:**
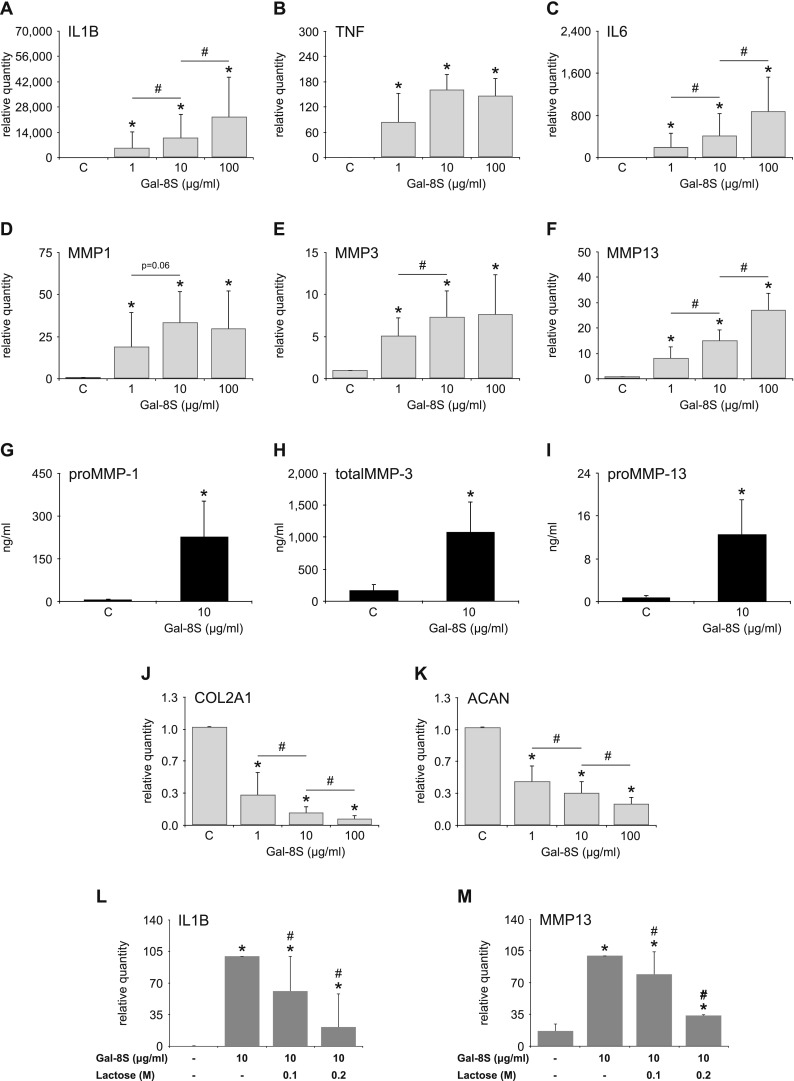
Glycan-inhibitable effect of Gal-8S on expression of OA-related marker genes and proteins. Chondrocytes of OA patients were starved overnight prior to treatment with three concentrations of Gal-8S for 24 h. Total RNA was isolated and cell culture supernatants were collected. **a**–**f**, **j**–**k** mRNA levels of IL1B (**a**), TNF (**b**), IL6 (**c**), MMP1 (**d**), MMP3 (**e**), MMP13 (**f**), COL2A1 (**j**) and ACAN (**k**) were determined using RT-qPCR (*n* = 6 patients). Results are expressed as relative quantities (mean ± SD) compared to untreated controls set to 1. *p < 0.05 (paired *t* test or Wilcoxon signed-rank test vs control). #*p* < 0.05 (paired *t* test or Wilcoxon signed-rank test). **g**–**i**) Concentrations (ng/ml) of proMMP-1 (**g**), total MMP-3 (**h**), and proMMP-13 (**i**) in cell culture supernatants of chondrocytes (*n* = 5 patients) treated with 10 µg/ml Gal-8S for 24 h, as determined by ELISA. *p < 0.05 (mean ± SD; paired *t* test vs control). **l**–**m** Chondrocytes (*n* = 6 patients) were treated with 10 µg/ml Gal-8S for 24 h in the absence or presence of 0.1 M or 0.2 M lactose. mRNA levels of IL1B (**l**) and MMP13 (**m**) were determined using RT-qPCR. Results are expressed as relative quantities (mean ± SD) compared to controls in the absence of cognate sugar set to 100. *p < 0.05 (paired *t* test vs control). #*p* < 0.05 (paired *t* test or Wilcoxon signed-rank test vs Gal-8S-treated cells)

**Fig. 4:**
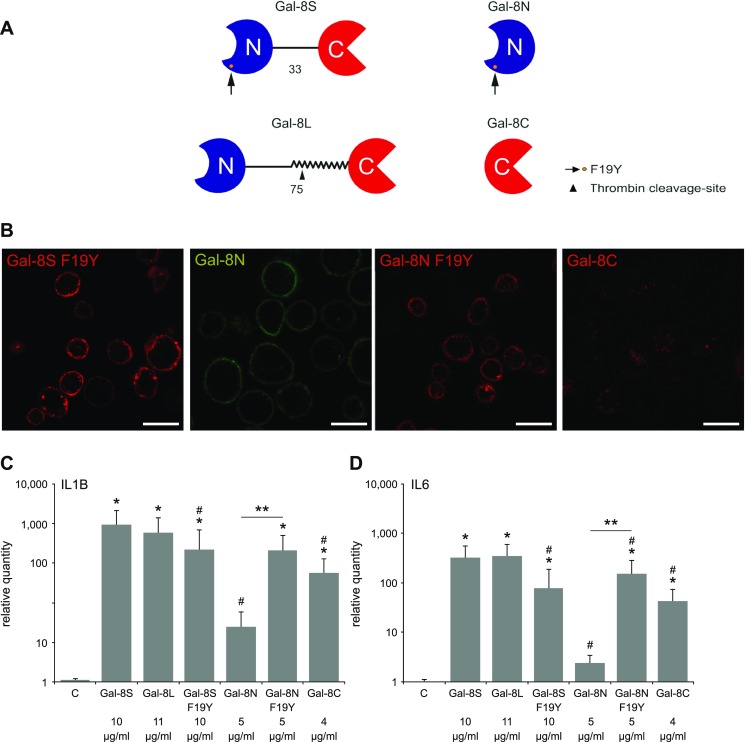
Activity of the 8L isoform, the SNP-based variant and the two separated Gal-8 domains (8 N, 8C) as inducers of functional disease markers in OA chondrocytes. **a** Schematic illustration of the molecular architecture, the site of the SNP-based alteration (arrow) and the position of the thrombin cleavage site in the long linker version (arrowhead). Cleavage will generate the separated CRDs, shown in the right part. **b** Cultured OA chondrocytes were trypsinized and resuspended prior to labeling with Gal-8S (F19Y)-AlexaFluor555 (red), Gal-8 N-FITC (green), Gal-8 N (F19Y)-AlexaFluor555 (red), or Gal-8C-AlexaFluor555 (red) at 4 °C. All proteins were used at 0.1 µg/µl. After 10 min of incubation, cells were washed and analyzed using laser scanning microscopy, with the focus plane set to the center of cells. Shown are representative results from chondrocytes of one patient (*n* = 3 patients). Scale bars: 20 μm. **c**, **d** Chondrocytes of five OA patients were starved overnight prior to 24 h treatment with 10 µg/ml Gal-8S or equimolar concentrations of Gal-8L, Gal-8S (F19Y), Gal-8 N, Gal-8 N (F19Y), and Gal-8C. mRNA levels of IL1B (**c**) and IL6 (**d**) were determined using RT-qPCR and expressed as relative quantities (mean ± SD) with respect to untreated controls set to 1. *p < 0.05 (Wilcoxon signed-rank test vs control). #*p* < 0.05 (Wilcoxon signed-rank test vs Gal-8S-treated cells). **p < 0.05 [Wilcoxon signed-rank test between data for Gal-8 N and Gal-8 N (F19Y)]

In assays with Gal-8S-treated and untreated cell cultures, Gal-8S was identified to be a potent inducer of functional disease markers such as IL1B, TNF, IL6, MMP1, MMP3 and MMP13. Significant changes were already seen at 1 µg/ml on the level of mRNA (Fig. 3a–f) and at 10 µg/ml on the level of secreted proteins (Fig. 3g–i). In agreement, apparent already at a concentration of 1 µg/ml, significant effects were observed for Gal-8S-induced downregulation of COL2A1 and ACAN expression (Fig. 3j, k). As shown for binding of fluorescent Gal-8S (Fig. 2a), the extent of measured responses was markedly diminished by the presence of lactose (Fig. 3l, m). The naturally abundant form of Gal-8 thus is a potent inducer of functional disease markers. Despite the low-level expression of the L form, additional experiments were performed to explore its activity comparatively. Additionally, the indications for clinical relevance of presence of the SNP-based F19Y variant in cohorts with autoimmune diseases, i.e., rheumatoid arthritis and myasthenia gravis [53], warranted analysis beyond the prevalent wild-type Gal-8S protein. To illustrate the mentioned structural features, Fig. 4a presents a schematic overview of the set of tested proteins.

### Effect of natural variants of Gal-8 on functional disease markers

In the first step of this comparative analysis, laser scanning microscopy was performed with the SNP-based F19Y variant protein and the two carbohydrate recognition domains (CRDs) of Gal-8 that can physiologically be generated by linker cleavage from the L form. It revealed binding of the F19Y variant and the two types of N-terminal domains [Gal-8 N and Gal-8 N (F19Y)] to the surface of OA chondrocytes, whereas binding of labeled Gal-8C was not observed at this concentration (Fig. 4b). When testing the effect of the set of full-length Gal-8 proteins [i.e., Gal-8S, Gal-8L, and Gal-8S (F19Y)] on the expression of IL1B and IL6 genes at equimolar concentrations, the obtained values for the two proteins with difference in linker length (i.e., Gal-8S and Gal-8L) were within the range of random fluctuations (Fig. 4c, d). In contrast, the occurrence of the SNP-based amino acid substitution resulted in a statistically significant decrease of induction of IL1B and IL6 genes. The free CRDs of the wild-type protein triggered markedly less (Gal-8 N) or less (Gal-8C) activity at this concentration, indicating bivalency of the prevalent form to be a structural factor to yield the strongest effect. Interestingly, the N-terminal CRD of the F19Y variant, but not that of the wild-type protein, maintained a level of activity comparable to that of the corresponding full-length protein (Fig. 4c, d). Both, the tandem-repeat type protein and the CRD harboring the F19Y substitution, are thus active: the full-length variant protein less than the full-length wild-type protein, its N-terminal CRD stronger than that of wild-type Gal-8. Linker cleavage will also generate the C-terminal CRD that maintains activity (Fig. 4c, d). The demonstration of the general effector capacity of Gal-8 on functional disease markers seen for all forms of this lectin, even on the level of the CRD, prompted to proceed with defining Gal-8′s impact on gene expression by a broad-scale approach.

### Gal-8S reprograms OA chondrocyte gene expression

Genome-wide analysis revealed the nature of impact of Gal-8S on the mRNA signature of articular chondrocytes (Supplementary File 7A–D). The list of the most upregulated genes prominently included a set of cytokines and chemokine ligands such as CXCL8, CCL20, IL6, CXCL3 and CXCL1 as well as inducible nitric oxide synthase 2 (NOS2) (Supplementary File 7A, B). Independent RT-qPCR experiments for five selected genes confirmed the array data (Supplementary File 7E). When setting the ranking of the 20 most upregulated genes in relation to respective results of our previously performed studies on Gal-1 and Gal-3 [17, 18], a high degree of similarity regarding the position of genes including those coding for CXCL8, CXCL3, CXCL1 and CXCL2 was found (upper part of Supplementary File 7C). It is intriguing to see that the profiles at the bottom part disclose a notable degree of supplementary entries (Supplementary File 7C). This phenomenon, however, occurs beyond these listed genes, as documented in Supplementary File 8 for glycogenes and Supplementary File 9 for genes that are likely to be involved in disease progression (e.g., MMPs). Notably, the gene for Gal-8 itself is upregulated by a factor of 1.9, suggesting an autoregulatory loop.

In addition to positive gene regulation, Gal-8S, like Gal-1 and -3, was capable to reduce mRNA production, as already indicated in Fig. 3j, k. Among the most downregulated genes, PDK4 and GREM1 appear to be of particular interest (Supplementary File 7D). PDK4 encodes a mitochondrial protein that acts as a regulator for shifting cellular energy utilization from glycolytic to fatty acid metabolism. It was previously found to rank among the most differentially regulated genes (with a fold-change of − 14.1) between OA and control cartilage [56]. GREM1, whose expression had been reported to be decreased by Gal-1 and Gal-3, too, in our previous studies [17, 18], is an antagonist of bone morphogenetic proteins in the TGF-β signaling pathway, which has been associated with chondrocyte hypertrophy in OA chondrocytes [57]. Additional downregulated genes are listed in Supplementary Files 8 and 9, again with evidence for overlapping and supplementing entries among the three galectins.

Following array profiling, bioinformatic analyses of the gene set upregulated by Gal-8S were performed to track down potential functional implications of the altered mRNA signature. Indeed, Metacore analysis connected Gal-8S activity to immune responses, inflammatory processes and diseases of connective tissues, joints and the musculoskeletal system (Supplementary File 10). Aiming to disclose the molecular switch used by Gal-8S, an additional set of bioinformatic tools was employed.

### Gal-8S-induced pro-degradative/inflammatory mRNA signature involves NF-κB

**Fig. 5:**
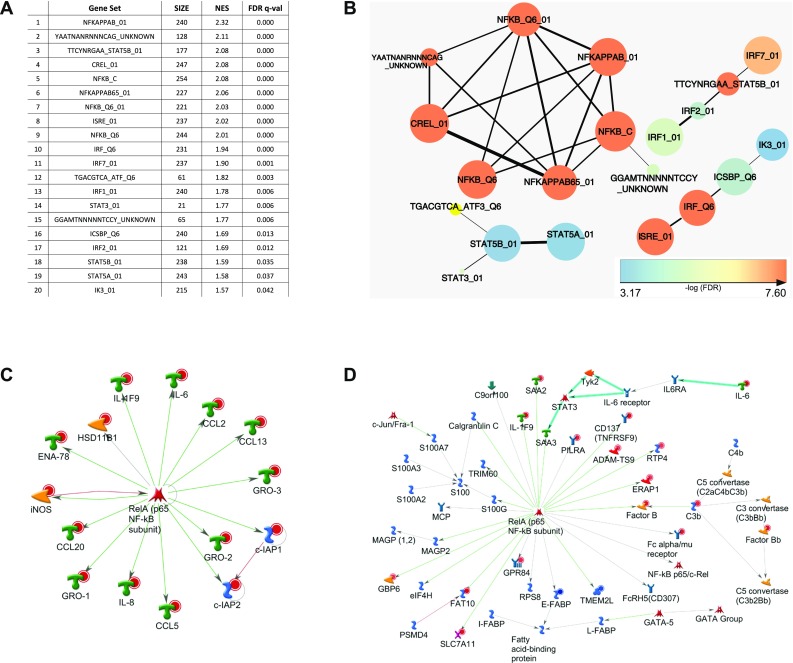
Bioinformatic analyses of genes with upregulated expression points towards a role of the NF-κB signaling pathway. **a** Top 20 results of GSEA against C3 tft (transcription factor target genes) v6.0 database in the order of normalized enrichment score (NES). *GS* gene set, *SIZE* size of the gene set, *FDR* false discovery rate. **b** Cytoscape enrichment map visualization of significant C3.tft v6.0 GSEA results. Nodes are colored according to FDR values (please see calibration scale, bottom left). Size of the nodes is proportional to the number of genes within each gene set. Line thickness indicates the extent of gene set overlap. **c** The 20 top regulated genes (according to |log2FC|-values) were subjected to Metacore’s transcription regulation algorithm, resulting in the scheme of the top scoring network according to the p value: ‘RelA p65 NF-κB subunit’. All genes presented were upregulated, as indicated by red circles. All connections shown correspond to fragments of canonical pathways. Colors of connections indicate types of interactions (as advised by Metacore): green: activation, red: inhibition, grey: unspecified. **d** The list of genes (|log2FC| > 2 and adjusted *p* < 0.05 including fold-changes and *p* values) was processed by the Metacore ‘generate network’ algorithm, allowing a maximum of 50 items per calculated network. The network ‘FAT10, Factor B, Fc α/μ receptor, ERAP1 and E–FABP’ is presented. The same color coding as in panel **c** applies to displaying genes and interactions

Using GSEA of the C3 transcription factor targets database, a notable overrepresentation of genes harboring sites for NF-κB binding was identified in the Gal-8S-induced gene set (Fig. 5a). This result was visualized using the Cytoscape enrichment map (Fig. 5b). As indicated by Metacore’s transcription regulation algorithm, the NF-κB subunit p65 was a central positive regulator for 15 of the 20 most upregulated genes by Gal-8S in OA chondrocytes (Fig. 5c). Participation of p65 to account for co-regulation of this mRNA signature was independently inferred using an alternative approach (Metacore’s ‘generate network’ algorithm). This technique analyzed the entire set of genes significantly affected by Gal-8S (|log2FC| > 2 and adjusted *p* < 0.05) leading to the scheme shown in Fig. 5d.

**Fig. 6:**
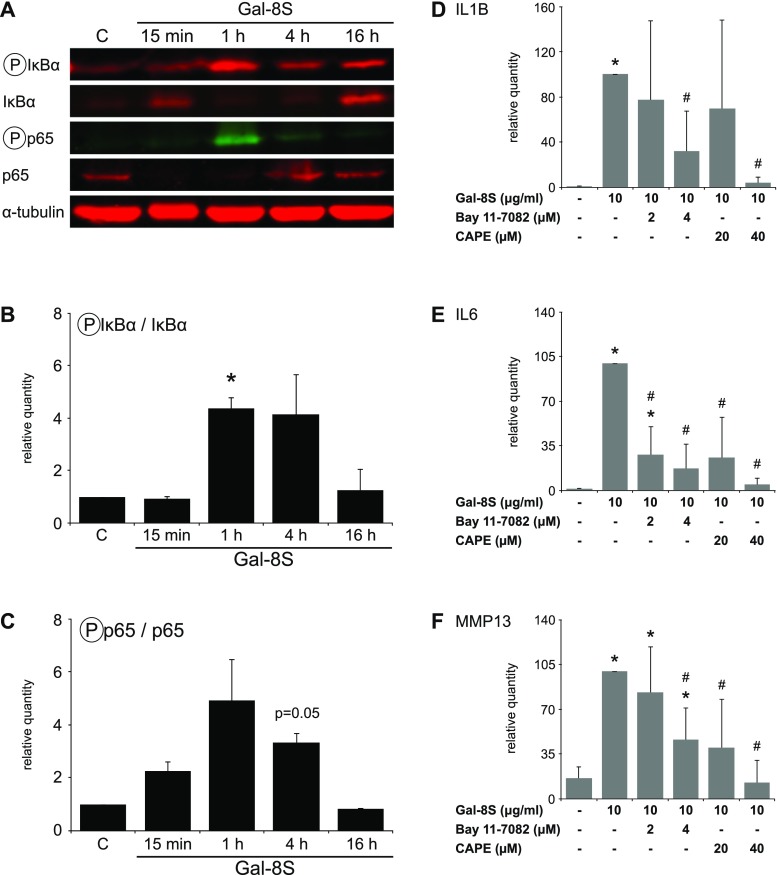
Gal-8 activity in OA chondrocytes is mediated by the NF-κB signaling pathway. **a**–**c** Quantitative WB analyses were performed with proteins isolated from OA chondrocytes after overnight starvation and treatment with 10 µg/ml Gal-8S for 15 min, 1, 4, and 16 h. **a** Shown are blots of one representative patient for phosphorylated IκBα, IκBα, phosphorylated p65, p65, and α-tubulin. **b**–**c** Over-time ratios are presented between phosphorylated IκBα and IκBα (**b**), as well as phosphorylated p65 and p65 (**c**). Data were normalized for α-tubulin and expressed as relative quantity compared to the untreated control set to 1 (*n* = 2 patients; *p < 0.05; paired *t* test vs untreated control). **d**–**f** Chondrocytes of four OA patients were starved overnight prior to 24 h treatment with 10 µg/ml Gal-8S in the absence or presence of NF-κB inhibitors. mRNA levels of IL1B (**d**), IL6 (**e**), and MMP13 (**f**) were determined via RT-qPCR. Results are expressed as relative quantities (mean ± SD) with respect to Gal-8S-treated chondrocytes set to 100. *p < 0.05 (paired *t* test vs untreated control). #*p* < 0.05 (paired *t* test vs Gal-8S-treated cells)

Taking the next step from these computationally obtained data, we experimentally studied the activation of key mediators of the NF-κB pathway by Gal-8S. Quantitative WB analyses showed that the phosphorylation of IκBα was significantly induced in a time-dependent manner with a peak at 1 h post-treatment of the chondrocytes (Fig. 6a, b). Corroborating this result, the phosphorylation of p65 followed a comparable kinetic pattern (Fig. 6a, c), leading to the availability of activated p65 for nuclear translocation. Extent of phosphorylation was reduced by lactose that precludes carbohydrate-dependent binding of Gal-8 as compared to the control value without exposure of cells to the lectin (data not shown). Blocking Gal-8S-induced phosphorylation of IκBα using the inhibitor Bay 11-7082 and impairing nuclear translocation of activated NF-κB using CAPE resulted in a dose-dependent reduction of Gal-8S-mediated transcription of IL1B, IL6 and MMP13 genes (Fig. 6d–f). These results are in line with the conclusion that canonical NF-κB signaling plays a key role in the gene-regulatory activity of Gal-8S in OA chondrocytes. Intriguingly, Gal-8S thus employs a similar downstream route as previously discovered for Gal-1 and -3 [17, 18].

In a broader context, the presented data argue in favor of functional cooperation between the three galectins. The emerging concept of a galectin network that shapes a pro-degradative/inflammatory microenvironment via NF-κB by functional cooperation of its constituents was subsequently tested by measuring the effect of galectins in combination, as they are present under pathophysiological conditions.

### Gal-8S functionally cooperates with Gal-1 and -3

**Fig. 7:**
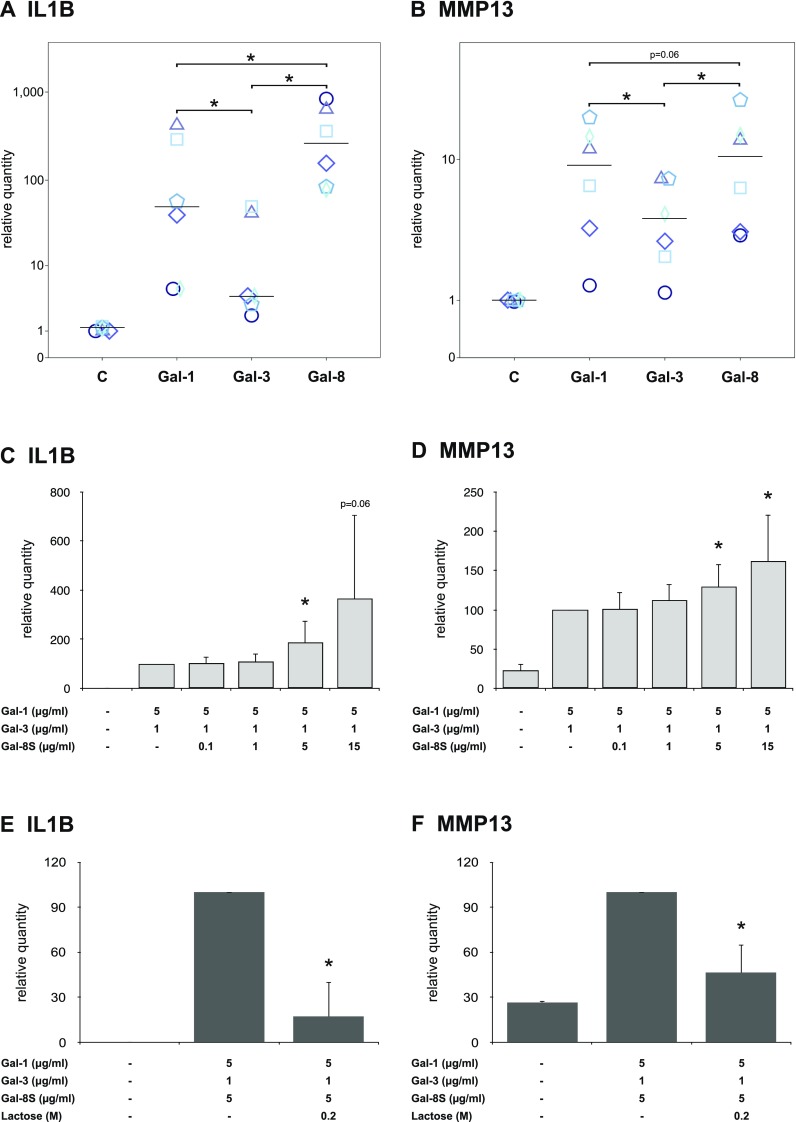
Functional cooperation between Gal-8, Gal-1 and Gal-3 in OA chondrocytes. **a**, **b** Chondrocytes of six OA patients were starved overnight prior to treatment with Gal-1 (10 µg/ml), Gal-3 (18 µg/ml) and Gal-8 (24 µg/ml) for 24 h. Total RNA was isolated and mRNA expression levels of IL1B (**a**) and MMP13 (**b**) were determined using RT-qPCR. Results are presented as dotplots showing values for cells of each patient as relative quantities with respect to untreated controls set to 1. The median values are indicated for Gal-1, -3, and -8 treatment. *p < 0.05 [Wilcoxon signed-rank test (panel **a**) or paired *t* test (panel **b**)]. **c**–**d** Chondrocytes of six OA patients were starved overnight prior to treatment with 5 µg/ml Gal-1 and 1 µg/ml Gal-3 together with increasing concentrations of Gal-8 for 24 h. Total RNA was isolated and mRNA levels of IL1B (**c**) and MMP13 (**d**) were determined using RT-qPCR. Results are expressed as relative quantities (mean ± SD) with respect to cells treated with 5 µg/ml Gal-1 and 1 µg/ml Gal-3 set to 100. The value for the untreated control in panel **c** is 0.3 ± 0.5. *p < 0.05 (paired *t* test vs cells treated with 5 µg/ml Gal-1 and 1 µg/ml Gal-3). **e**, **f** Chondrocytes of three OA patients were treated with a mixture of 5 µg/ml Gal-1, 1 µg/ml Gal-3, and 5 µg/ml Gal-8 for 24 h in the absence or presence of 0.2 M lactose. Total RNA was isolated and mRNA levels of IL1B (**e**) and MMP13 (**f**) were determined using RT-qPCR. Results are expressed as relative quantities (mean ± SD) with respect to cells treated with 5 µg/ml Gal-1, 1 µg/ml Gal-3, and 5 µg/ml Gal-8S set to 100. The value for the untreated control in panel E is 0.3 ± 0.2. *p < 0.05 (paired *t* test vs cells treated with 5 µg/ml Gal-1, 1 µg/ml Gal-3, and 5 µg/ml Gal-8S)

When cultures of OA chondrocytes were stimulated in parallel with Gal-8S, -1 and -3, significant upregulation of IL1B and MMP13 transcription was consistently seen in each case (Fig. 7a, b; *p* < 0.05). For both transcripts, Gal-8S proved to be a potent effector, along with Gal-1 and, albeit being less active, with Gal-3. In independent experimental series using binary mixtures of either Gal-1 or -3 with Gal-8, where the subsaturating concentrations of 5 µg/ml Gal-1 and 1 µg/ml Gal-3 were kept constant, addition of Gal-8 to reach a concentration of 5 µg/ml resulted in significant increases of IL1B and MMP13 gene transcription in most cases (data not shown). By testing Gal-8S in a mixture with Gal-1 and -3 at these subsaturating concentrations, the question on functional cooperation was addressed. As shown in Fig. 7c, d, the addition of increasing Gal-8S quantities could cause significant enhancement of levels of IL1B- and MMP13-specific mRNAs, respectively. In both cases, a subsaturating concentration of 5 µg/ml Gal-8S was sufficient to elicit this effect. Of note, presence of cognate sugar abolished the combined activity of Gal-8S, Gal-1, and Gal-3 on IL1B and MMP13 gene transcription (Fig. 7e, f). On the level of the protein, a significantly enhanced 3.3 ± 3.1-fold protein secretion (*p* = 0.043; Wilcoxon; *n* = 5 OA patients) was measured in the case of MMP13. The three galectins can, therefore, functionally cooperate and trigger their activity in a carbohydrate-inhibitable manner.

## Discussion

Tracking down molecular switches in OA pathogenesis has the potential to lead to the development of innovative treatment modalities. Our present results identify Gal-8 as a clinically relevant factor in OA progression. Its expression in chondrocytes is correlated with the severity of cartilage degeneration, reaching a percentage of about 60% positivity at the highest MS. The secretion of Gal-8 by cultured OA chondrocytes, as it is the case for human Gal-1 and -3 in this cell system [17, 18], fulfills a key prerequisite for Gal-8 to exert functionality via auto- and paracrine mechanisms. The combination of secretion and subsequent cell surface binding is a means for growth regulation by galectins, and, indeed, “a significant fraction of the secreted Gal-8 remains bound to the extracellular surface” in the case of lung cancer cells [58], from here also affecting tumor cell migration [59]. In case of the cancer cells, Vinik et al. recently concluded that “the evidence, gathered thus far, implicates Gal-8 as a driver of a ‘vicious cycle’, whereby cancer cells that overexpress and secrete Gal-8 benefit from its potential to promote their own growth; potentiate epithelial–mesenchymal transition and induce secretion of metastasis-promoting agents at the metastatic niche that induce further recruitment and seeding of cancer cells. Further in-depth studies related to its mode of action are expected to support ongoing efforts aimed at implementing Gal-8-targeted therapies for the treatment of cancer patients” [60], an aim becoming a perspective for research on osteoarthritis by the data presented herein. The documented positive correlation between the MS score and immunopositivity is the prerequisite for such a driving-force role of galectin-8. Cultures of OA chondrocytes showed lactose-inhibitable binding of Gal-8-, and this binding triggered enormous effects on the profile of gene transcription, on protein production and eventually on the extent of secretion of a panel of functional OA markers. In particular, Gal-8 (mostly Gal-8S in situ) thereby contributed to shape a pro-degradative/inflammatory microenvironment.

On the level of Gal-8 structure, this activity does not appear to be markedly modulated by the length of the linker, because the 8L form was similarly active when compared to Gal-8S. The SNP-based amino acid exchange in the N-terminal CRD, on the other hand, decreased the activity of the variant protein. This substitution at position 19 had previously been revealed to attenuate Gal-8’s capacity as bridging molecule between exosome-like vesicles or as hemagglutinin [36], but to increase the extent of Gal-8-dependent inhibition of tumor cell proliferation [61]. This finding encourages performing correlation analysis of SNP occurrence in OA patients. Intriguingly, the free N-terminal CRD of this variant still harbors gene expression-activating ability. In other words, cleavage of the linker of the variant by a protease, as documented for Gal-8L by thrombin [62] (see also Fig. 4a), will likely not harm the variant protein’s lowered activity, although Gal-8 (F19Y) has then lost its bivalency. A tendency for formation of di- or oligomers in the case of its N-terminal CRD on a cell surface, which could actually be trapped in solution in the case of the wild-type protein by a chemical cross-linker [41], may underlie this result (and the band at the respective position of a Gal-8S dimer in WB analysis of extracts shown in Supplementary File 2C; interestingly, WBs of synovial fluid of nine patients presented a signal for “a Gal-8 isoform of 69 kDa, in variable amounts” [40]). Such a behavior might also explain Gal-8 N’s efficiency to serve as substratum for lung cancer cells [58] and neutrophil adhesion [45], to activate platelets [46] and to co-stimulate antigen-specific T cell responses in the presence of antigen-presenting cells [44]. Looking at the variant’s crystal structure, the presence of tyrosine 19’s hydroxyl group caused a series of movements in the region of the N-terminus, especially involving residues 11–15, and the contribution to the thermodynamic balance sheet differed, too, between wild-type and variant proteins [61]. These changes may explain the impact of the amino-acid substitution on self-interaction when present at high local density on a cell surface, making counterreceptor binding and cross-linking in situ possible.

Operating as effector in OA chondrocytes, Gal-8S engages the NF-κB pathway as downstream signaling route. This also appears to be the case in a different cell system, i.e. when Gal-8 stimulates production of pro-inflammatory chemo- and cytokines such as IL-6, CXCL1, GM-CSF and RANTES in human microvascular endothelial (HMEC-1, HMVEC-L) cells [47, 63]. In this cell type, Gal-8S synthesis and secretion were stimulated by LPS and the signal for phosphorylation of the p65 subunit of NF-κB was most intense after 10 min of Gal-8S (2 µM) treatment [47]. Of note, Gal-8 herewith joins Gal-1 and -3 as broad-scale regulator via NF-κB in OA chondrocytes (not detected in non-OA diseases serving as controls), the three galectins sharing binding capacity as shown in Fig. 2b. In addition to an overlap, Gal-8 has its own aspects within the pro-degradative/inflammatory activation profile, each of the three galectins making its contribution. Functional cooperation is thus not simple redundancy but can have supplementing character. As such, occurrence of specific responses to Gal-8 in the framework of otherwise redundant activity profiles of Gal-1 and -8 was also documented for murine splenic B cells, where both galectins (at 1 mM) induced IL-10, but only Gal-8 led to an increase of IL-6 [48].

The presented evidence for congruence of effector routes and overlapping profiles of gene activation after concomitant upregulation of the three galectins in chondrocytes in the course of disease progression prompted to extend the previously started search for shared sequences in promoter regions and introns as putative binding sites for transcription factors [18]. Proceeding from presenting the genomic organization for Gal-1, -3 and -8 (Supplementary File 11), experimental information on the transcription start site for the Gal-8 gene (Supplementary File 12) is given. Based on this result, a list of computationally detected sites with ligand potential in the Gal-8 promoter region and introns was established (Supplementary File 13), allowing identification of a series of sequence motifs and possibly reactive transcription factors common for the three galectins (Supplementary File 14). This list gives direction for respective efforts towards characterizing the nature of concerted galectin upregulation.

Of particular note for the in vivo situation (here, other cell types likely add to the interrelationship within the inflammatory process), we not only revealed considerable overlap among the three induced expression profiles by testing each protein alone, but we documented the cooperation of Gal-1, -3 and -8 by testing mixtures. In OA chondrocytes, the expression of these three galectins is correlated with the MS, and the three proteins cooperate to establish pathophysiological aspects most likely relevant for disease progression. Our analysis with focus on OA chondrocytes, therefore, establishes an illustrative precedent for functional teamwork among the three galectins which differ in modular architecture, i.e., tandem-repeat type (Gal-8), chimera type (Gal-3) and proto type (Gal-1). This teaches a salient lesson, i.e., to consider galectin functionality in a network with varying outcomes. In fact, when comparing this situation to the currently few cases of documented intra-network cross-talk, it becomes clear that generalizations are not allowed. On murine naïve peripheral CD4 T cells, the interrelationship in the galectin system is drastically different: only Gal-8 elicits proliferation, whereas Gal-3 antagonizes the co-stimulatory effects of Gal-8 that is also triggered by Gal-1 at considerably higher concentration [64]. The observation that Gal-8-dependent neutrophil adhesion and proMMP-9 processing cannot be mediated by Gal-1 and -3 [45], while Gal-3 can favor or inhibit osteoclastogenesis depending on the context [65–68], making positive or negative effects on Gal-8 activity possible [49], attest the inherent complexity of the interplay of galectins in an obviously site-dependent manner. As a branch of the activity profile of the tumor suppressor p16^INK4a^, Gal-1 and -3 expressions are stringently directed into opposite directions. In detail, anti-apoptotic Gal-3 is an antagonist of the pro-anoikis effector Gal-1, while reprogramming of glycosylation and an increase of the Gal-1 counterreceptor α_5_-integrin enhanced cellular susceptibility to Gal-1 en route to eventually initiate caspase 8-dependent anoikis [69–71]. Regarding subsequent investigations, the comparative characterization of functional counterreceptors for the galectins in OA chondrocytes will warrant further efforts. In this respect, Gal-8 has been reported to associate exclusively with distinct membrane constituents, especially members of the integrin family such as CD11a/CD11b or α_5_β_1_-integrins, the C-type mannose macrophage receptor and MMP-9 in different cell systems [43, 45, 49, 58, 72, 73], indicating the lectins’ selectivity for functional pairing with a small set of glycoproteins from the broad glycoproteomic diversity of a cell. Even more, soluble glycoproteins in the medium deserve attention, too, as the instructive example of the neutralizing effect of a variant of CD44 and fibrin(ogen) on Gal-8’s pro-apoptotic activity on synovial fluid cells in rheumatoid arthritis attests [40].

In summary, our study identified Gal-8 as a potent factor in OA pathogenesis, acting in concert with Gal-1 and -3. The detected functional cooperation underscores the emerging paradigmatic concept to consider galectin expression as a network with context-dependent characteristics. Analysis of the broad-scale NF-κB-involving alterations of gene expression revealed overlapping patterns with characteristic features, directing interest towards further data processing, the search for elicitors of galectin upregulation and the nature of counter receptors. Obviously, the next step to take is to complete the identification of the galectin network in OA. Whether Gal-4, which appears to be upregulated together with Gal-1, -3, -8 in OA [16], is also an active part of this network, is currently under investigation. Hereby, we attempt to move closer to the first comprehensive functional cross-talk analysis for galectins in a widely occurring disease, providing a role model for future studies on the clinical relevance of galectins.

## Materials and methods

### Galectins and antibodies

Human Gal-8 proteins, as compiled in Fig. 4a, were obtained by recombinant production and purified by affinity chromatography of lactose-presenting resin as crucial step, using one- and two-dimensional gel electrophoresis and gel filtration to ascertain purity [36, 59, 61]. Labeling by commercial fluorescent dyes was performed under activity-preserving conditions as described [52]. The antibody preparation was checked by systematic ELISAs for cross-reactivity against other human galectins, and cross-reactive material was removed chromatographically using protein-loaded beads [28, 30, 31].

### Clinical specimens

Human articular cartilage specimens for histological analyses and cell culture assays were obtained from OA patients during total knee replacement surgery with written informed consent and in accordance with the terms of the ethics committee of the Medical University of Vienna (EK-No. 1065/2011).

### Histological assessment

Cartilage specimens from 15 OA patients (8 female, 7 male; age range 51–81) were selected macroscopically to allow immunohistochemical analysis of a wide range of degeneration stages. Paraffin sections of patient’s femoral condyles were stained with Safranin O [16] to enable a grading of cartilage degeneration according to the Mankin Score. In total, 120 regions (5–9 regions per patient) were graded. Consecutive tissue sections were processed for immunohistochemical staining using anti-Gal-8 antibody.

### Immunohistochemistry

Immunohistochemical staining for Gal-8 presence was performed as previously described [16]. In brief, deparaffinized cartilage tissue sections were incubated with rabbit polyclonal antibody against human Gal-8. Staining was developed using horseradish peroxidase containing reagent (VECTASTAIN Elite ABC Kit, Vector Labs) and 3,3′-diaminobenzidine tetrahydrochloride hydrate (Fluka) and H_2_O_2_ as substrates. Sections were counterstained using Mayer’s hemalum solution (Merck). As described [15], the percentages of Gal-8-positive chondrocytes in the regions of interest were assessed by two independent observers.

### Immunofluorescence detection of Gal-8 in cartilage tissue

As previously described, Gal-8 detection in tissue slices of human OA cartilage was performed using immunofluorescence staining [18]. In brief, after blocking, slides were incubated overnight at 4 °C with rabbit polyclonal anti-Gal-8 antibody (1 µg/ml). On the following day, slides were incubated for 1 h at room temperature with AlexaFluor555-anti-rabbit (1:750; Life Technologies) together with 3 µg/ml DAPI (Sigma-Aldrich) and finally mounted for microscopy. Images were taken using the LSM700 laser scanning microscope (Carl Zeiss) at 630× magnification.

### Fluorescence detection of galectin-binding sites in cartilage tissue

The detection of Gal-8S binding sites in cartilage tissue was performed as previously described using fluorescence staining [18]. In brief, slides were processed for fluorescence staining and incubated overnight at 4 °C with AlexaFluor488-conjugated Gal-8S (10 µg/ml) together with 3 µg/ml DAPI, or AlexaFluor555-conjugated Gal-8S with AlexaFluor488-labeled Gal-1, or AlexaFluor555-conjugated Gal-8S with AlexaFluor488-labeled Gal-3, or AlexaFluor488-labeled Gal-1 with AlexaFluor555-conjugated Gal-3. The next day, slides were mounted for microscopy and images were obtained using the LSM700 laser scanning microscope (Carl Zeiss) at 630× magnification.

### Cell culture

Human OA chondrocytes were isolated from femoral condyles and tibial plateaus and cultured following established protocols [14, 17]. For all assays, primary cells were used without subculturing to preserve the chondrocyte phenotype. Chondrocyte cultures (90% confluent) were serum-starved overnight and treated with recombinant human galectins. Moreover, chondrocytes were treated with recombinant Gal-8S or in combinations with recombinant Gal-1 and Gal-3 in presence or absence of lactose. NF-κB pathway components were inhibited using Bay 11-7082 (Merck) and CAPE (Merck), which were added 1 h prior to stimulation with recombinant Gal-8S. In addition, to induce pro-inflammatory conditions, chondrocytes were stimulated with IL-1β, TNF-α or IL-8 (all from Biolegend). Please find time periods of treatment or concentrations directly in the figures or in the figure legends.

### Fluorescence staining of galectin-binding sites on the surface of viable chondrocytes

As previously described [18], chondrocytes grown as monolayer culture were trypsinized to obtain a cell suspension of 3 × 10^5^ cells in 50 µl PBS. Cells were incubated with 5 µg/50 µl AlexaFluor488-labeled Gal-8S in presence or absence of 0.1 M lactose, or combinations of 5 µg/50 µl AlexaFluor488-labeled Gal-8S and 2 µg/50 µl AlexaFluor555-labeled Gal-1 or 5 µg/50 µl AlexaFluor488-labeled Gal-8S and 4 µg/50 µl AlexaFluor555-labeled Gal-3 or 2 µg/50 µl AlexaFluor488-labeled Gal-1 and 4 µg/50 µl AlexaFluor555-labeled Gal-3, or with 5 µg/50 µl of either AlexaFluor555-labeled Gal-8S (F19Y), FITC-labeled Gal-8 N, AlexaFluor555-labeled Gal-8 N (F19Y), or AlexaFluor555-labeled Gal-8C. Images were immediately taken without fixation using laser scanning microscopy (LSM700, 630× magnification). In some cases, z stack fluorescence images were captured across the diameter of cells followed by reconstruction of 3D images. Then, 3D movies of stained chondrocytes were generated with Zen software following object manipulation in 3D space.

### Transmission electron microscopy

Starved and Gal-8S-treated as well as untreated cell cultures were fixed with 2.5% glutaraldehyde and 2% PFA, post-fixed with Palade’s buffered osmium tetroxide and embedded in Epon according to standard protocols. 1 µm semi-thin sections were stained with toluidine blue and imaged under a ZEISS light microcope (AxioImager.A2) equipped with a ZEISS AxioCam MRc5 color camera. 70 nm ultra-thin sections were contrasted with lead citrate and uranyl acetate before being imaged with an FEI Tecnai20 electron microscope equipped with a 4 K Eagle-CCD camera. Images were processed with Adobe Photoshop.

### RT-qPCR

Total RNA isolation, cDNA synthesis and SYBR-green-based RT-qPCR experiments were performed as previously described [14]. In brief, total RNA extraction was performed using innuPREP RNA Mini Kit (Analytik Jena). Each RNA sample was examined for quality and quantity on NanoDrop 1000 and Agilent 2100 Bioanalyzer prior to reverse transcription into cDNA. RNA integrity numbers were between 9.4 and 10. The protocols followed the minimal guidelines for the design and documentation of qPCR experiments [74]. A detailed checklist containing all relevant information is provided by the authors upon request. mRNA levels were calculated as relative quantities compared to the untreated controls considering amplification efficiencies and normalization to succinate dehydrogenase complex, subunit A (SDHA) which had been identified as stable reference gene under the experimental conditions of this study.

### Detection of LGALS8 splice variants in OA chondrocytes

Four alternatively spliced transcript variants of LGALS8 encoding two different isoforms are known. Transcript variants 1 and 4 encode the longer isoform Gal-8L, whereas variants 2 and 3 encode the shorter isoform Gals-8S. Essentially, variants 1 and 4 differ from variants 2 and 3 by an additional exon of 126 base pairs (bp). Based on a recently described method [75], we designed three primer pairs using Primer3 software [76] that flanked the spliced region, allowing simultaneous detection of two distinguishable amplicons for each primer pair, with each amplicon specific for the longer or shorter variant, respectively (Supplementary File 2A). Using these primers and RNA from OA chondrocytes, RT-qPCR was performed as described above. Afterwards, electrophoretic separation of RT-qPCR products was performed in 1.5% agarose gel with GelRed as dye for visualization.

### Microarray

Human OA chondrocytes were isolated from five female patients (56–76 years). Cells were cultured in 25 cm^2^ flasks. Following overnight serum starvation, cells were incubated with 10 µg/ml Gal-8S for 24 h. Control of RNA quality and quantity was performed as described above (A260/A280: 1.98–2.07; RNA integrity numbers: 9.6–10). Gene chip analysis was performed as previously described [17] using 200 ng RNA per sample. The microarray data of Gal-8S-treated chondrocytes discussed in this publication will be publicly available through Gene Expression Omnibus (GEO) database upon acceptance of the manuscript. Data from Gal-3 and Gal-1-treated chondrocytes were reproduced from GEO database (accession numbers: Gal-3: GSE85254, Gal-1: GSE68760).

### Bioinformatic analyses

Expression data were RMA-normalized on the basis of probe sets [77]. Next, data were filtered using the knnCV method within the R genefilter [78] package. The R limma package [79] was used to process data on probe set level, yielding a list of probe sets sorted by ascending adjusted *p* values (Bonferroni–Holm). The list was truncated for |log_2_FC| > 2 and adjusted *p* < 0.05. Probe sets were remapped to gene symbols based on the PrimeView v3.6 annotation file, and by selecting for each symbol that probe set which exhibited largest |log_2_FC| between Gal8-treatment and control. Next we took the top 20 regulated symbols according to |log2FC|-values, all of which were upregulated, and imported into Metacore software.

### Detection of transcription start points

Total RNA was isolated from human colon cancer (DLD-1) and prostate cancer (PC-3) cells with the RNeasy Mini-Kit (Qiagen). Using the GeneRacer Kit (Thermofisher), the RNAs were prepared stepwise to allow detection of their 5′ ends by fusing a specific 5′GeneRacer Oligo to the mRNAs and reverse transcribe them into cDNAs with SuperScript III Reverse Transcriptase and Random Primers (provided by the GeneRacer Kit). RACE-ready (Rapid Amplification of cDNA Ends) cDNA is processed in PCR by using the GeneRacer 5′Primer and a reverse primer specific for Gal-8 (hGal8-GSP-RACE-5; GCTTGGGTACTTTGTAAGTCCGAGCTGA), following a nested-PCR with GeneRacer-nested 5′ Primer and hGal8-GSP-RACE-4 (CCTGGAATCTGTCTGCGTCACTAGGAACA). The resulting fragments were cloned into the TOPO-vector pCR4 (Thermofisher), to followed by transformation of cells of the *E. coli* strain TOP10. Plasmids were later purified using a commercial plasmid kit (Stratec Biomedical) and then sequenced (GATC).

### Analysis of promoter and intron regions

The proximal promoter region (− 2500 bp) and the 9 introns of the gene of human Gal-8 were processed by the MatInspector software (Matrix Library 10.0) with setting that exclude low-quality, as done for the cases of Gal-1 and -3 ensuring direct comparability [17, 18].

### Elisa

Gal-8 levels were determined in cell culture supernatants of chondrocytes cultured under pro-inflammatory conditions using ELISA, using internal blank controls (Sigma-Aldrich). To achieve detectable concentrations, supernatants were concentrated using centrifugal filter units (Amicon Ultra, Ultracel 3 K membrane, Merck Millipore). The levels of proMMP-1, proMMP-13 and totalMMP-3 were detected in cell culture supernatants of Gal-8S-treated chondrocytes (MMP ELISAs from R&D Systems). Supernatants of untreated chondrocytes served as controls. Standard curve ranges were 0.164–40 ng/ml for Gal-8, 0.156–10 ng/ml for proMMP-1 and totalMMP-3, and 78–5000 pg/ml for proMMP-13.

### Quantitative Western blot

Following previously established protocols [17, 18], proteins were extracted from OA chondrocytes for quantitative Western blot analyses. In brief, cells were lysed; proteins were separated using 10% acrylamide gels and transferred to a nitrocellulose membrane. After blocking with 5% milk/PBS, membranes were incubated for 2 h with primary antibodies: anti-Gal-8 (rabbit polyclonal), anti-phospho IκBα (Ser32/36, 1:1000, mouse monoclonal; Cell Signaling), anti-IκBα (1:1000, rabbit monoclonal; Cell Signaling), anti-phospho NF-κB p65 (Ser536; 1:1000; rabbit monoclonal; Cell Signaling), anti-NF-κB p65 (1:1000; mouse monoclonal; Cell Signaling) and anti-α-tubulin (1:1000; mouse monoclonal; Cell Signaling). Thereafter, membranes were incubated for 1 h with DyLight 800 nm-labeled goat anti-rabbit IgG (1:15,000; Thermo Scientific) and IRDye 680LT goat anti-mouse IgG (1:15,000; Licor). The immunoreactive protein bands were detected and quantified using the Odyssey Imager CLx (Licor). The ratios between phospho IκBα and IκBα, as well as phospho p65 and p65 (all normalized for α-tubulin) were calculated as relative quantities in comparison to the untreated controls set to 1.

### Statistical analyses

Correlation analysis between Gal-8-immunopositivity and cartilage degeneration (represented by the Mankin score) was performed using SPSS 24.0. Pearson’s correlation coefficients were calculated for each patient separately, and Wilcoxon signed-rank test was performed to test whether the median correlation coefficient was different from 0. Statistical analyses of RT-qPCR, ELISA and quantitative Western blot data were performed using SPSS 24.0. Normal distribution of the data was analyzed using the Shapiro–Wilk test. Statistical significance of the data was delineated using paired *t* test (normally distributed data) or Wilcoxon signed-rank test (non-normally distributed data).

## Electronic supplementary material

Below is the link to the electronic supplementary material.

**Supplementary File 1: Specificity control in immunohistochemical detection.** Serial sections of a specimen of an OA patient were processed by routine IHC with (A) and without (B) the incubation step with anti-Gal-8-specific antibodies. Illustrations are presented at two different levels of magnifications (40x, 400x), excluding any antigen-independent signal generation. Scale bars at 40x magnifications: 500 µm. Scale bars at 400x magnifications: 100 µm (PDF 519 kb)

**Supplementary File 2: Isoform detection of Gal-8 by RT-qPCR and WB.** (A) Schematic illustration of the location of three primer pairs (F1-F3) that were designed to span the additional exon region that distinguishes the long (L) isoform (transcript variants 1 and 4) from the standard short (S) isoform (transcript variants 2 and 3) of LGALS8. The lengths of expected amplicons are given in base pairs (bp). The primer pairs were designed by tools to achieve specificity for the four transcript variants. (B) Agarose gel electrophoretic analysis of RT-qPCR products amplified using the primer sets listed in panel A. Note that in all three cases the primer pairs amplified only one product that corresponds to the length of the shorter predicted amplicon. (C) WB analysis was performed with extracts from OA chondrocytes (n = 3 patients), one representative result being shown. Recombinant Gal-8S (300 ng; molecular weight: 35.8 kDa) and Gal-8L (150 ng; molecular weight: 40.3 kDa) were used as positive controls. Positions of molecular weight marker bands at 75 kDa, 50 kDa, and 37 kDa are shown (PDF 157 kb)

**Supplementary File 3: Gal-8 and its binding sites localize in chondrons of OA cartilage.** (A-B) OA cartilage sections were processed (A) with or without (A) an IgG fraction against Gal-8 followed by immunofluorescence detection using AlexaFluor555-labeled second-step antibodies (red) or (B) with Gal-8S-AlexaFluor488 (green) together with DAPI (blue) prior to analysis using laser scanning microscopy. Differential interference contrast (DIC) imaging was included. Scale bar: 20 μm. (C-E) OA cartilage sections were processed with (C) Gal-8S-AlexaFluor555 (red) and Gal-1-AlexaFluor488 (green), (D) Gal-8S-AlexaFluor555 (red) and Gal-3-AlexaFluor488 (blue), or (E) Gal-1-AlexaFluor488 (green) and Gal-3-AlexaFluor555 (blue) prior to analysis using laser scanning microscopy. Documentation of tissue structure by DIC imaging is included. Scale bar: 20 µm (PDF 2257 kb)

**Supplementary File 4: Localization of binding sites for fluorescent Gal-8 at 4** **°C.** Cultured OA chondrocytes were trypsinized and resuspended prior to labeling with Gal-8S-AlexaFluor488 (green) at 4 °C. After 10 min of incubation, cells were washed and analyzed using laser scanning microscopy. A series of 16 images was recorded at 1 μm intervals to create a stack in the Z axis. Shown is the projection from the Z stack generated using ZEN software (MOV 1741 kb)

**Supplementary File 5: Localization of binding sites for fluorescent Gal-8 at 37** **°C.** Cultured OA chondrocytes were trypsinized and resuspended prior to labeling with Gal-8S-AlexaFluor488 (green) at 37 °C. After 10 min of incubation, cells were washed and analyzed using laser scanning microscopy. A series of 16 images was recorded at 1 μm intervals to create a stack in the Z axis. Shown is the projection from the Z stack generated using ZEN software (MOV 1578 kb)

**Supplementary File 6: Cellular morphology of control and Gal-8S-treated primary chondrocytes.** OA chondrocytes were starved overnight and were treated either with 10 µg/ml Gal-8S overnight (right side) or were left untreated (left side). Representative microphotographs are shown. (A) Toluidine Blue-stained Sects. (1 µm) of pelleted cells. The color balance was adjusted with Adobe Photoshop. Scale bars: 50 µm. Insets show a higher magnification of the specimens (scale bars: 20 µm). (B) Transmission electron microphotographs of ultrathin Sects. (70 nm). Arrows point to Golgi or ER, respectively. Scale bars: 0.5 µm (PDF 2089 kb)

**Supplementary File 7: Microarray analysis identifying the 20 most up- and downregulated genes in Gal-8S-treated OA chondrocytes.** Chondrocytes of five OA patients (numbered with “1”–“5”) were starved overnight prior to treatment with 10 µg/ml Gal-8S for 24 h. (A) Heat maps of RMA-normalized log_2_-expression values for the 20 most upregulated and the 20 most downregulated genes were generated following microarray analysis and ranked according to ascending fold-change values. (B) For the cases of upregulation, the fold-changes of mRNA levels in Gal-8S-treated versus untreated chondrocytes across all five patients were calculated. The adjusted p-values are also given. (C) The ranking of the 20 most upregulated Gal-8S-induced genes is compared to respective rankings seen for Gal-1 and -3. (D) Fold-changes of mRNA levels in Gal-8S-treated versus untreated chondrocytes in cases of downregulation across all five patients were calculated. The adjusted p-values are also given. (E) Results of the microarray experiments were ascertained using RT-qPCR analysis in the same RNA samples as used in the microarray analysis. Data are presented as log_2_-expression values. For each gene checked, all five treated samples yielded higher levels of expression than control samples, resulting in a *p* value of 0.031 (Wilcoxon signed-rank test) for each gene (PDF 200 kb)

**Supplementary File 8: Comparison of the sets of glycogenes regulated by Gal-8S, Gal-3 or Gal-1 as determined by microarray analysis.** The ratios between mRNA levels of glycogenes in Gal-8S-, Gal-3- or Gal-1-treated versus untreated chondrocytes as well as p-values, corrected for multiple hypothesis testing by the Benjamini–Hochberg method, are given. Data from Gal-3 and Gal-1-treated chondrocytes were reproduced from GEO (accession numbers: Gal-3: GSE85254, Gal-1: GSE68760) (PDF 74 kb)

**Supplementary File 9: Comparison of the sets of genes relevant for degradative and inflammatory processes regulated by Gal-8S, Gal-3, or Gal-1 as determined by microarray analysis.** The ratios between mRNA levels of OA relevant genes in Gal-8S-, Gal-3- or Gal-1-treated versus untreated chondrocytes as well as p-values, corrected for multiple hypothesis testing by the Benjamini–Hochberg method, are given. Data from Gal-3 and Gal-1-treated chondrocytes were reproduced from GEO (accession numbers: Gal-3: GSE85254, Gal-1: GSE68760) (PDF 67 kb)

**Supplementary File 10: Bioinformatic analysis of canonical pathways, process networks and diseases affected by Gal-8S treatment.** Shown are the results of Metacore’s compare experiments algorithm. (A-C) Orange bars indicate genes induced by Gal-8S. (A) Top 20 scored canonical pathway maps. (B) Top 20 scored process networks. (C) Top 20 scored diseases (PDF 833 kb)

**Supplementary File 11: Genomic organization of the genes for human Gal-1, -3 and -8.** The organization of the genes of human Gal-1 (NM002305.3), Gal-3 (NM002306.3; NM001177388.1) and Gal-8 (NM201545.2; NM006499.4; NM201543.2; NM201544.2) are shown schematically. All variants are used that have a consolidated status in the NCBI gene database. Exons are given as white (non-coding sequence) or grey (coding sequence) boxes. The numbers of base pairs are entered above the respective boxes, with the size of the coding sequence in brackets if the exon contains a non-coding region. Introns are depicted as black lines, with corresponding sizes given below lines. For overview, the sizes of the exons and introns of the Gal-8 gene were only shown if there is a change in base pairs. Translation start points are marked by ATG, whereas the transcription start points (TSPs) are marked by an arrow. The TSPs were chosen based on literature data (TIFF 176 kb)

**Supplementary File 12: Transcription start points (TSPs) of the gene for human Gal-8.** Shown is the 5´ UTR region of the human Gal-8 on genomic level. The translation start point is denoted by ATG. (A) The exact position of each of the known TSP (arrows) is given in brackets as distance in basepairs relative to ATG. TSPs were given in the literature or detected experimentally via GeneRACER (Rapid amplification of cDNA ends) position c was found in the human PC-3 cell line, positions a, d, e, f, g in the human DLD-1 cell line. In both cell lines, TSP b, too, was found. Literature data report presence of two transcripts whose origin is the site labeled by (*), differing by a deletion of the indicated 343 bp-long sequence. The indicated 1906 bp-long sequence is not present in transcripts starting from the sites denoted as a-e. (B) The sequence for the 5´ UTR of human Gal-8 is given, the positions of the different TSPs (*, a-g) being marked by black triangles. The positions on the right are in relation to the translation start, i.e. ATG (bold, underlined). Grey-shaded sequences indicate areas that are not represented in transcripts (see A) (TIFF 424 kb)

**Supplementary File 13: MatInspector-based detection of putative transcription factor binding sites in promoter and intron regions of the gene of human Gal-8.** Shown are the relative positions (*Pos.*) of potential binding motifs for transcription factors in relation to the transcription start point +1 (TSP). The analysis includes as well the promoter region 2500 bp upstream of the TSP as the introns downstream of the ATG. The location of the motif is either on the sense (+) or antisense (-) strand (Orientation). Sequence shows the corresponding DNA sequence, wherein the potential transcription factor binding site was found. The core sequence of the motif is indicated in capital letters. For analysis the core similarity was set to 1.0, which means a perfect match for the highest conserved region of the motif. The matrix similarity gives information about the score for the comparison with the respective matrix. In the course of this each position of the potential motif is compared with the highest conserved nucleotide at the same position in the matrix. The matrices with a score beyond 0.8 are shown in the table and clustered for their functional related families. Names of matrix families are given below the table (XLS 750 kb)

**Supplementary File 14: Compilation of computationally detected putative binding sites for transcription factors (TFs) in the promoter and intron regions of the genes for human Gal-1, -3 and -8.** The analyzed regions were -2000 to +97 for Gal-1, -2500 for Gal-3 and -2500 for Gal-8 (promoter regions) and the sequences of the introns of the genes of Gal-1 (3 introns), -3 (5 introns) and -8 (9 introns). A list of all detected TFs for either Gal-1 or Gal-3 was published previously [17, 18]. The putative binding sites were compared regarding to their shared or specific occurrence in the promoter and intron regions of the galectins (PDF 105 kb)

## Abbreviations

ACAN: Aggrecan
CAPE: Caffeic acid phenethyl ester
CCL: CC chemokine ligand
COL2A1: Collagen, type II, α1
CRD: Carbohydrate recognition domain
CXCL: CXC chemokine ligand
CXCR: CXC chemokine receptor
DAPI: 4′,6-diamidino-2-phenylindole
DIC: Differential interference contrast
ER: Endoplasmic reticulum
FITC: Fluorescein isothiocyanate
Gal: Galectin
GEO: Gene expression omnibus
GREM1: Gremlin 1
GSEA: Gene set enrichment analysis
IκB: Inhibitor of κB
IKK: IκB kinase
IL: Interleukin
LPS: Lipopolysaccharide
MMP: Matrix metalloproteinase
MS: Mankin score
NF-κB: Nuclear factor-κB
NOS2: Nitric oxide synthase 2
OA: Osteoarthritis
PBS: Phosphate-buffered saline
PDK4: Pyruvate dehydrogenase kinase 4
RANKL: Receptor activator of NF-κB ligand
RMA: Robust multichip average
SNP: Single nucleotide polymorphism
TGF: Transforming growth factor
TNF: Tumor necrosis factor
TSPs: Transcription start points
WB: Western blot
