# Supplementary figures and images for: Galectin-8 induces functional disease markers in human osteoarthritis and cooperates with galectins-1 and -3
Source: Cell Mol Life Sci. 2018 Jun 22;75(22):4187–205. doi: 10.1007/s00018-018-2856-2 (PMC6182346; doi:10.1007/s00018-018-2856-2)

Supplementary File 1

A

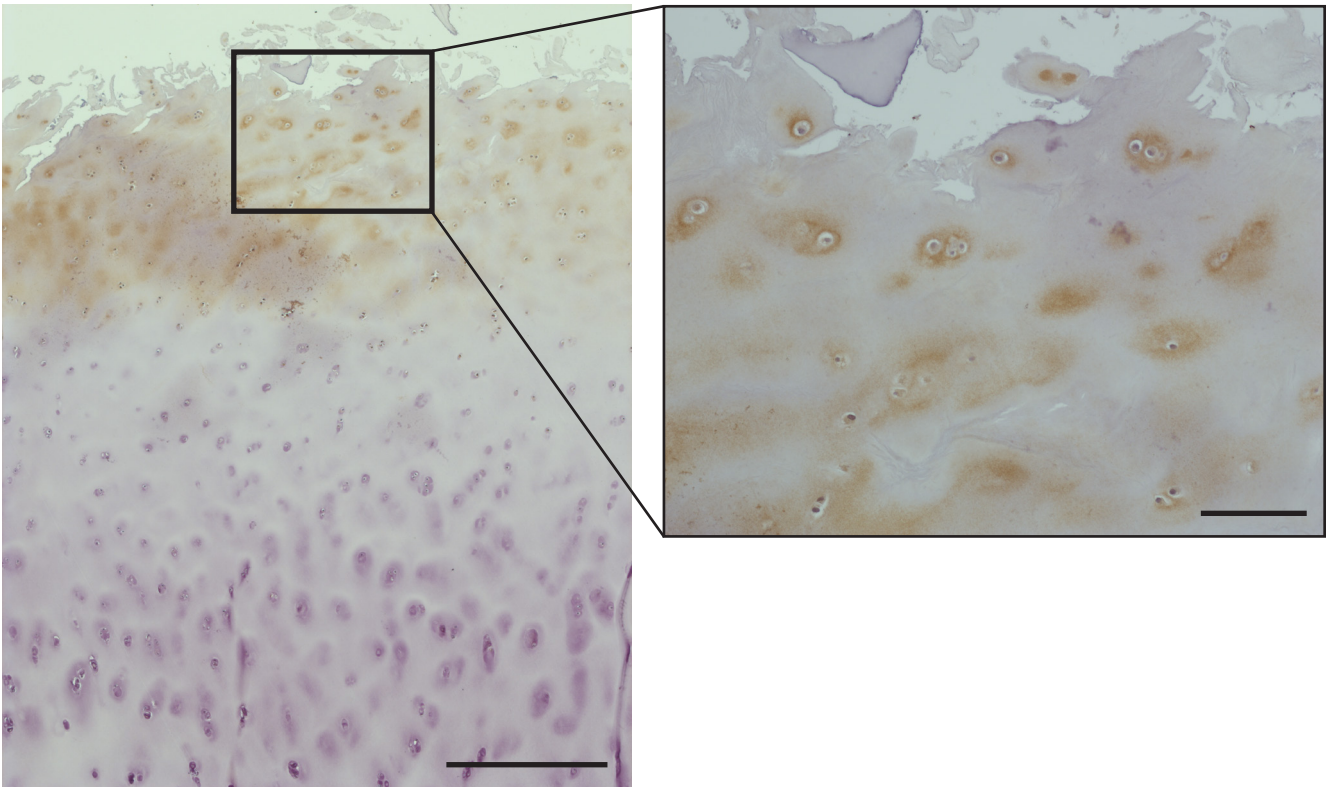

B

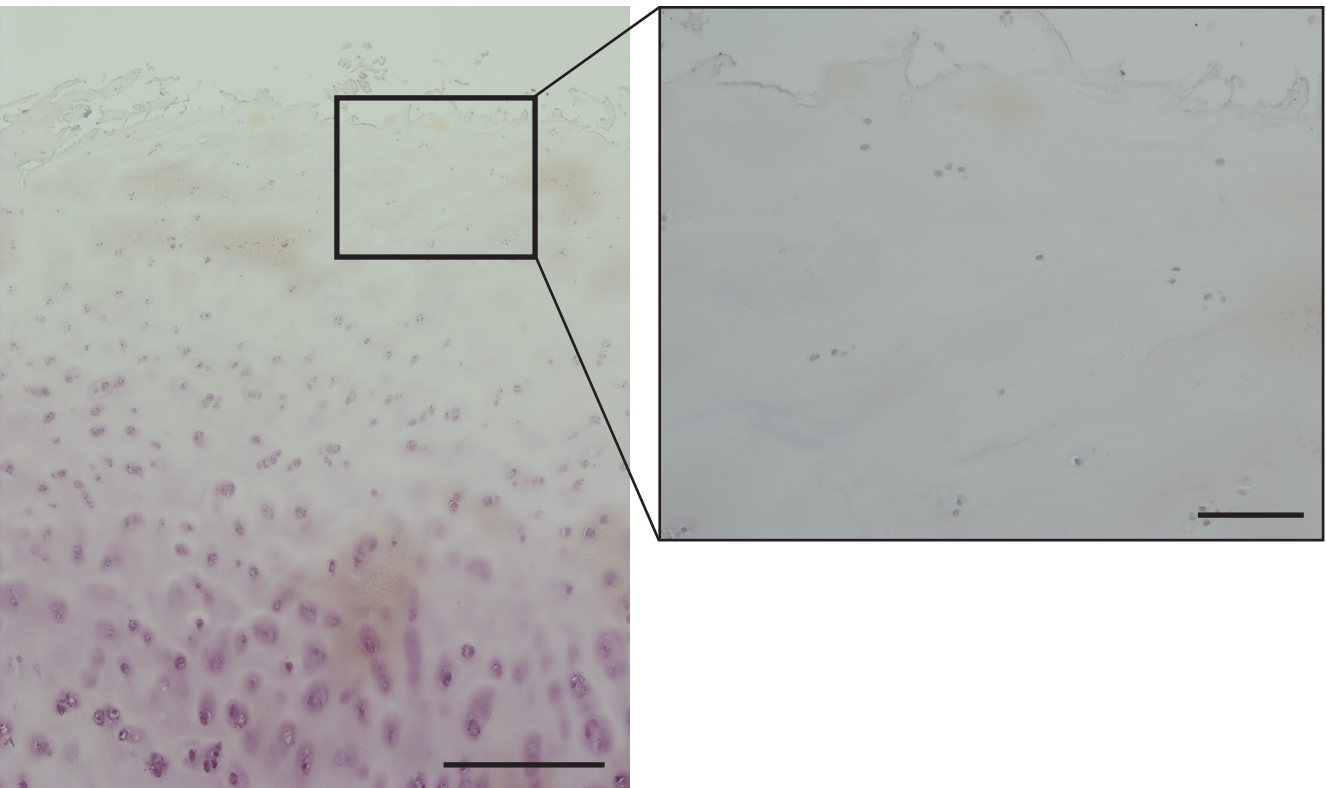

Supplement: Supplementary file 1 — Specificity control in immunohistochemical detection. Serial sections of a specimen of an OA patient were processed by routine IHC with (A) and without (B) the incubation step with anti-Gal-8-specific antibodies. Illustrations are presented at two different levels of magnifications (40x, 400x), excluding any antigen-independent signal generation. Scale bars at 40x magnifications: 500 µm. Scale bars at 400x magnifications: 100 µm (PDF 519 kb) [file 18_2018_2856_MOESM1_ESM.pdf]

# Supplementary File 2

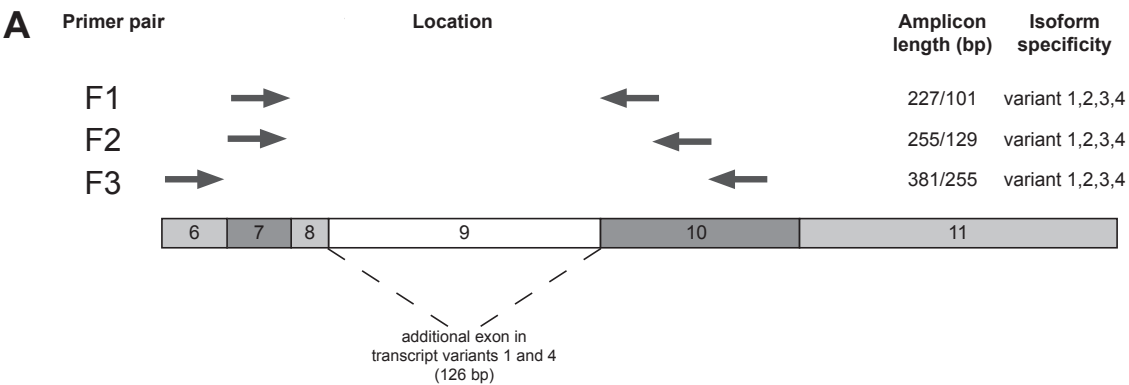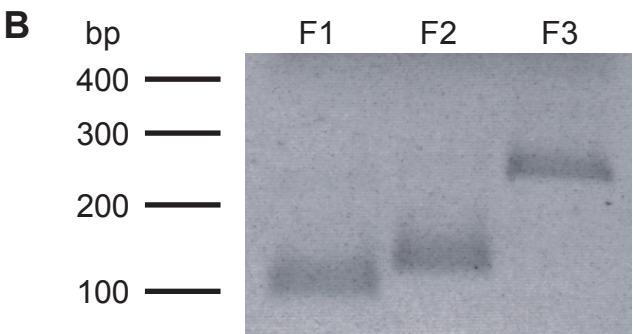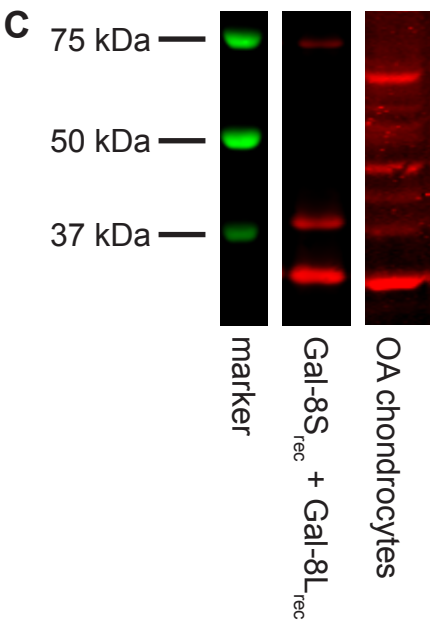

Supplement: Supplementary file 2 — Isoform detection of Gal-8 by RT-qPCR and WB. (A) Schematic illustration of the location of three primer pairs (F1-F3) that were designed to span the additional exon region that distinguishes the long (L) isoform (transcript variants 1 and 4) from the standard short (S) isoform (transcript variants 2 and 3) of LGALS8. The lengths of expected amplicons are given in base pairs (bp). The primer pairs were designed by tools to achieve specificity for the four transcript variants. (B) Agarose gel electrophoretic analysis of RT-qPCR products amplified using the primer sets listed in panel A. Note that in all three cases the primer pairs amplified only one product that corresponds to the length of the shorter predicted amplicon. (C) WB analysis was performed with extracts from OA chondrocytes (n = 3 patients), one representative result being shown. Recombinant Gal-8S (300 ng; molecular weight: 35.8 kDa) and Gal-8L (150 ng; molecular weight: 40.3 kDa) were used as positive controls. Positions of molecular weight marker bands at 75 kDa, 50 kDa, and 37 kDa are shown (PDF 157 kb) [file 18_2018_2856_MOESM2_ESM.pdf]

Supplementary File 3

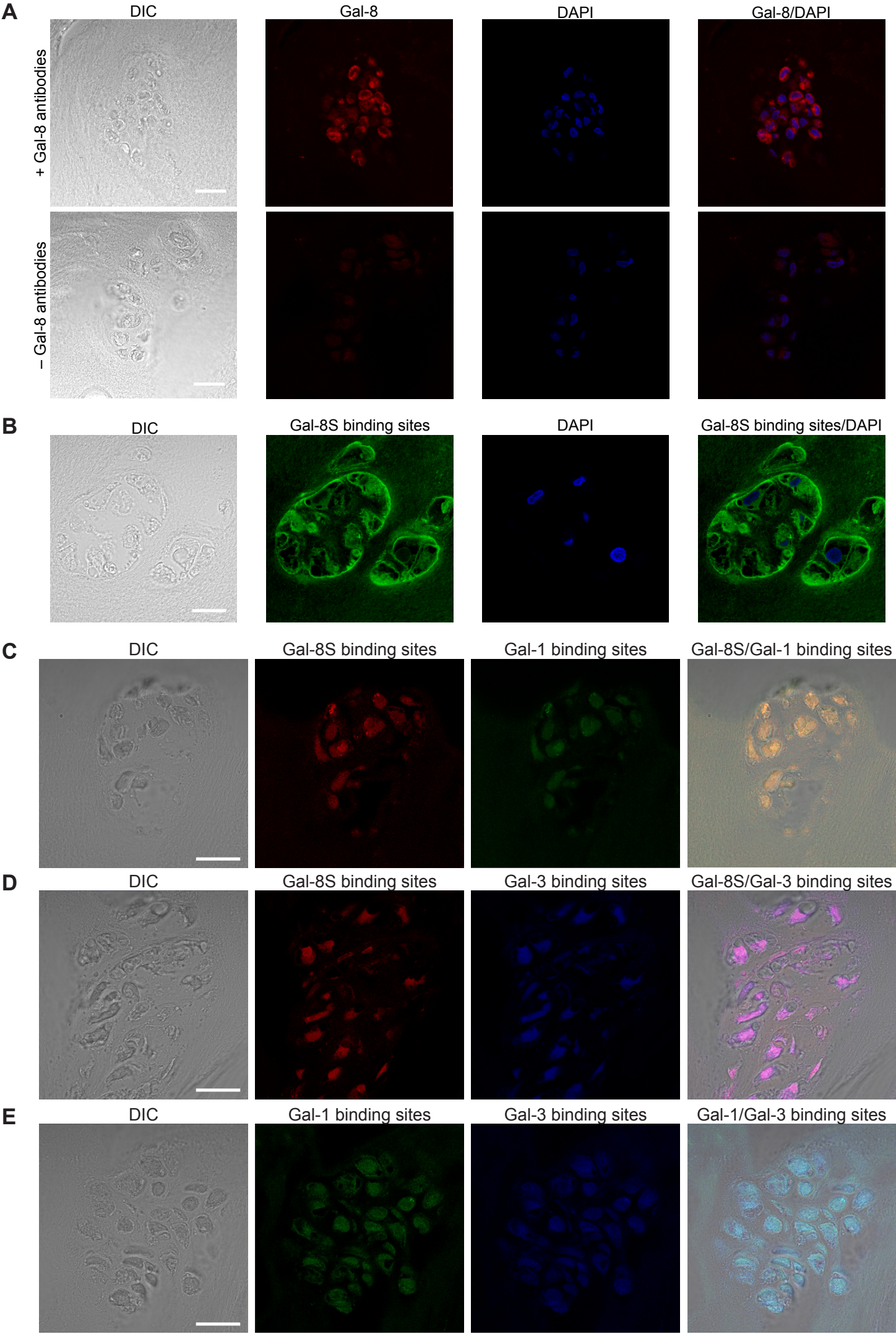

Supplement: Supplementary file 3 — Gal-8 and its binding sites localize in chondrons of OA cartilage. (A-B) OA cartilage sections were processed (A) with or without (A) an IgG fraction against Gal-8 followed by immunofluorescence detection using AlexaFluor555-labeled second-step antibodies (red) or (B) with Gal-8S-AlexaFluor488 (green) together with DAPI (blue) prior to analysis using laser scanning microscopy. Differential interference contrast (DIC) imaging was included. Scale bar: 20 μm. (C-E) OA cartilage sections were processed with (C) Gal-8S-AlexaFluor555 (red) and Gal-1-AlexaFluor488 (green), (D) Gal-8S-AlexaFluor555 (red) and Gal-3-AlexaFluor488 (blue), or (E) Gal-1-AlexaFluor488 (green) and Gal-3-AlexaFluor555 (blue) prior to analysis using laser scanning microscopy. Documentation of tissue structure by DIC imaging is included. Scale bar: 20 µm (PDF 2257 kb) [file 18_2018_2856_MOESM3_ESM.pdf]

Supplementary File 6

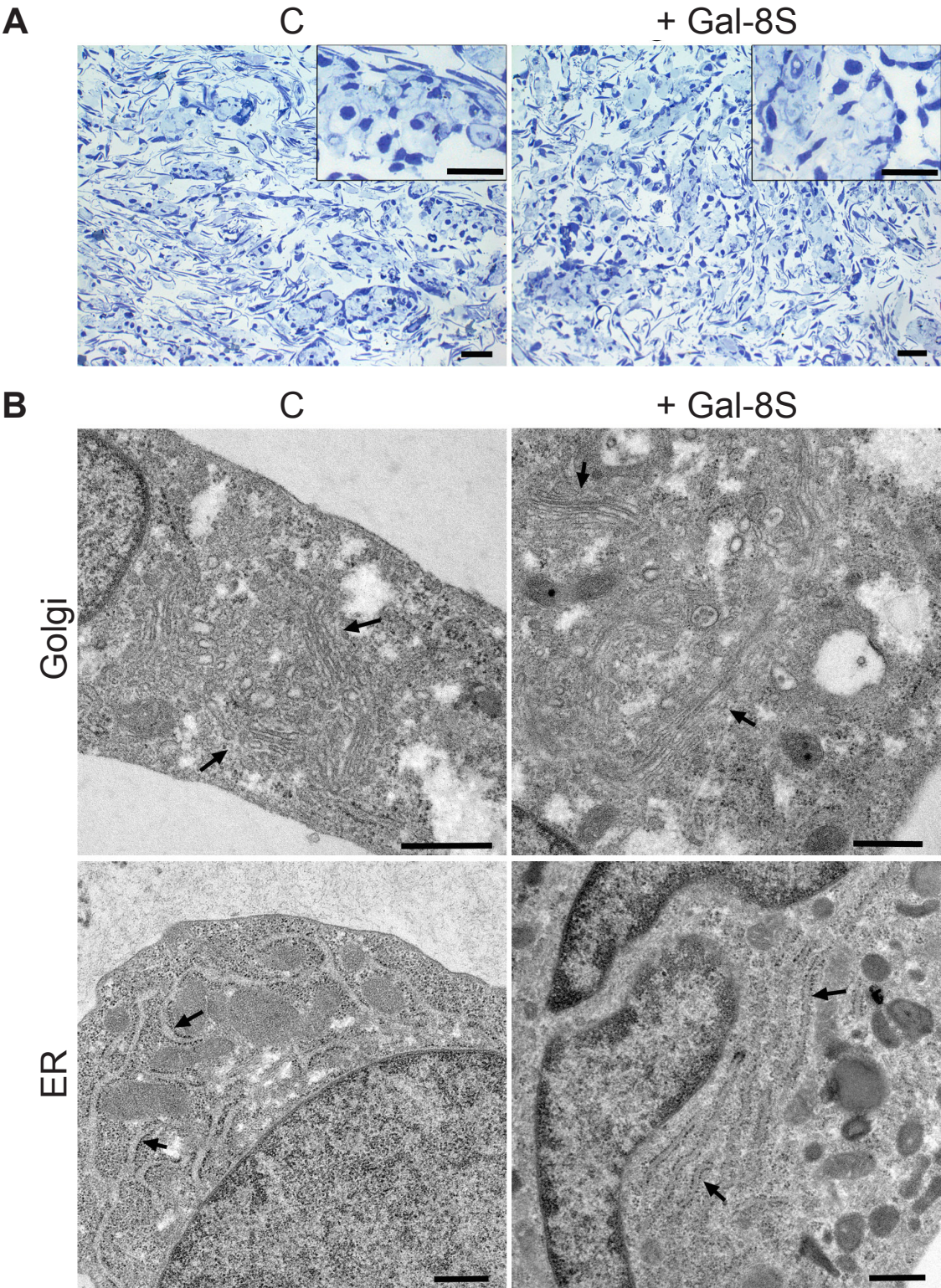

Supplement: Supplementary file 6 — Cellular morphology of control and Gal-8S-treated primary chondrocytes. OA chondrocytes were starved overnight and were treated either with 10 µg/ml Gal-8S overnight (right side) or were left untreated (left side). Representative microphotographs are shown. (A) Toluidine Blue-stained Sects. (1 µm) of pelleted cells. The color balance was adjusted with Adobe Photoshop. Scale bars: 50 µm. Insets show a higher magnification of the specimens (scale bars: 20 µm). (B) Transmission electron microphotographs of ultrathin Sects. (70 nm). Arrows point to Golgi or ER, respectively. Scale bars: 0.5 µm (PDF 2089 kb) [file 18_2018_2856_MOESM6_ESM.pdf]

Supplementary File 7

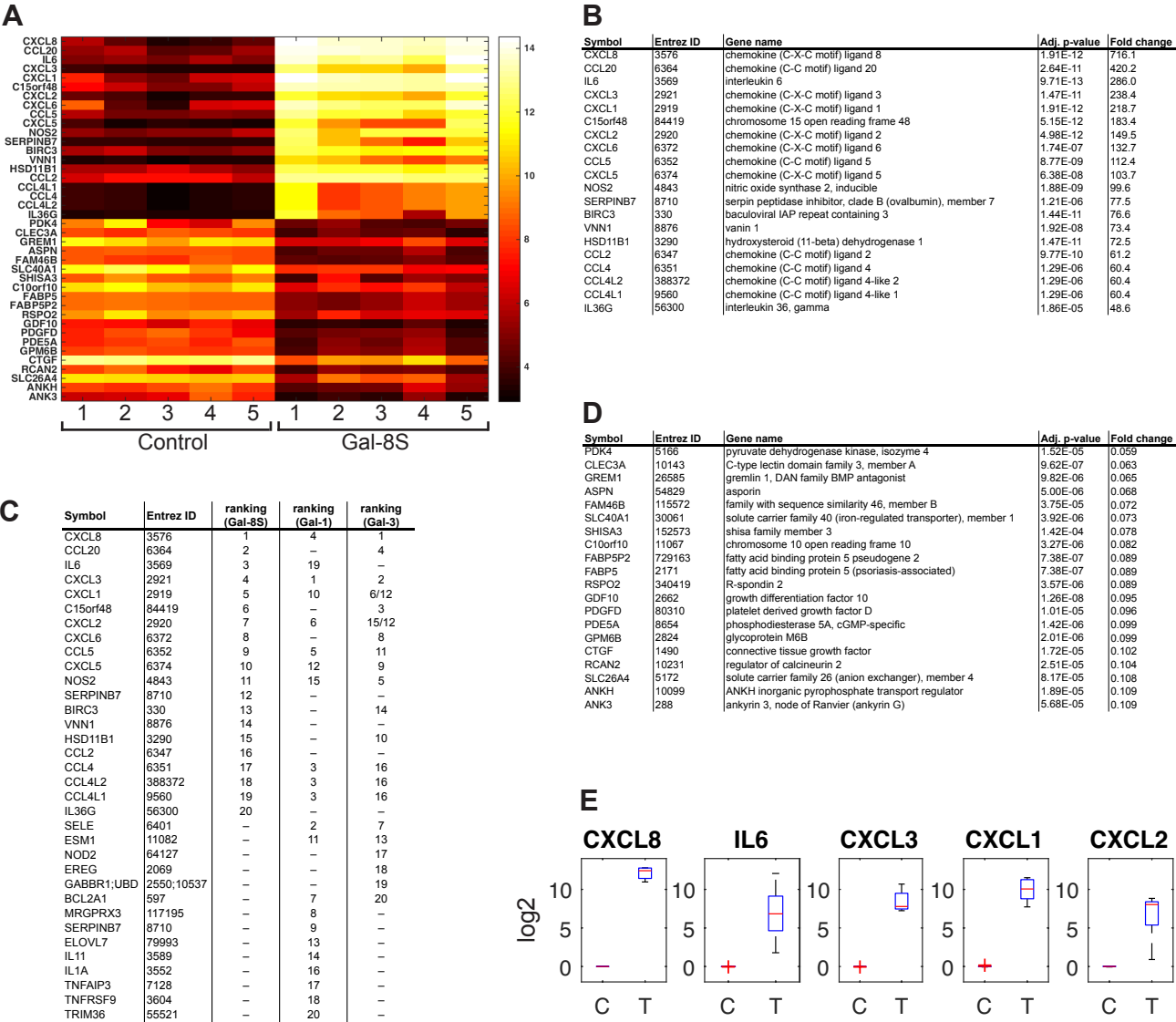

Supplement: Supplementary file 7 — Microarray analysis identifying the 20 most up- and downregulated genes in Gal-8S-treated OA chondrocytes. Chondrocytes of five OA patients (numbered with “1”–“5”) were starved overnight prior to treatment with 10 µg/ml Gal-8S for 24 h. (A) Heat maps of RMA-normalized log2-expression values for the 20 most upregulated and the 20 most downregulated genes were generated following microarray analysis and ranked according to ascending fold-change values. (B) For the cases of upregulation, the fold-changes of mRNA levels in Gal-8S-treated versus untreated chondrocytes across all five patients were calculated. The adjusted p-values are also given. (C) The ranking of the 20 most upregulated Gal-8S-induced genes is compared to respective rankings seen for Gal-1 and -3. (D) Fold-changes of mRNA levels in Gal-8S-treated versus untreated chondrocytes in cases of downregulation across all five patients were calculated. The adjusted p-values are also given. (E) Results of the microarray experiments were ascertained using RT-qPCR analysis in the same RNA samples as used in the microarray analysis. Data are presented as log2-expression values. For each gene checked, all five treated samples yielded higher levels of expression than control samples, resulting in a p value of 0.031 (Wilcoxon signed-rank test) for each gene (PDF 200 kb) [file 18_2018_2856_MOESM7_ESM.pdf]

Supplementary File 10

A

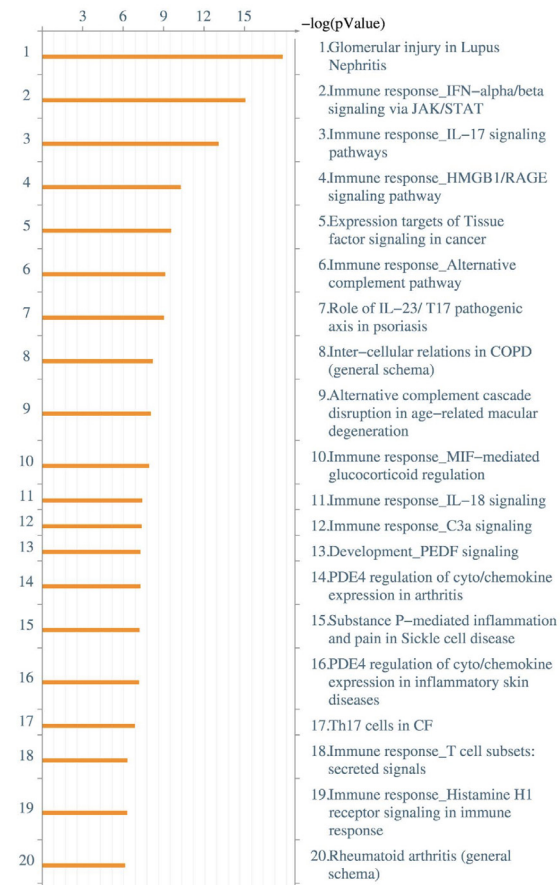

B

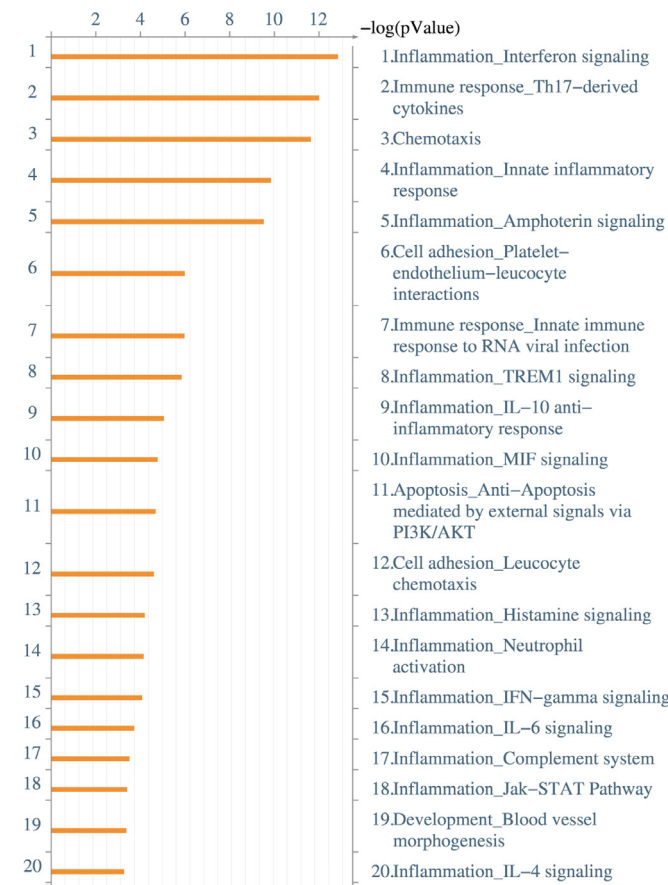

C

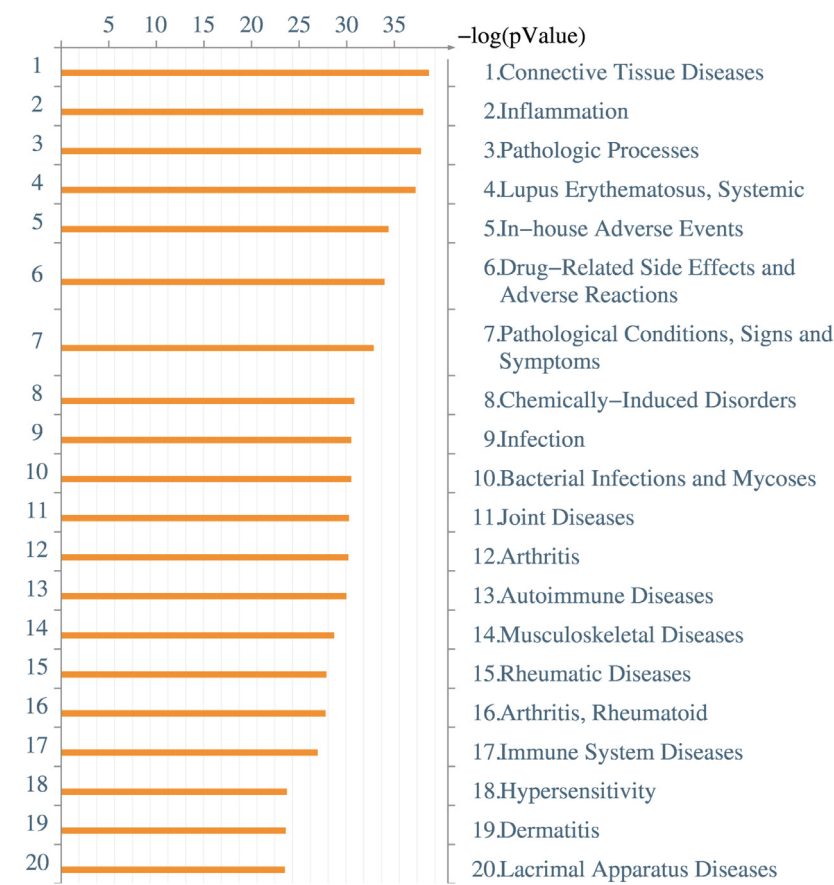

Supplement: Supplementary file 10 — Bioinformatic analysis of canonical pathways, process networks and diseases affected by Gal-8S treatment. Shown are the results of Metacore’s compare experiments algorithm. (A-C) Orange bars indicate genes induced by Gal-8S. (A) Top 20 scored canonical pathway maps. (B) Top 20 scored process networks. (C) Top 20 scored diseases (PDF 833 kb) [file 18_2018_2856_MOESM10_ESM.pdf]

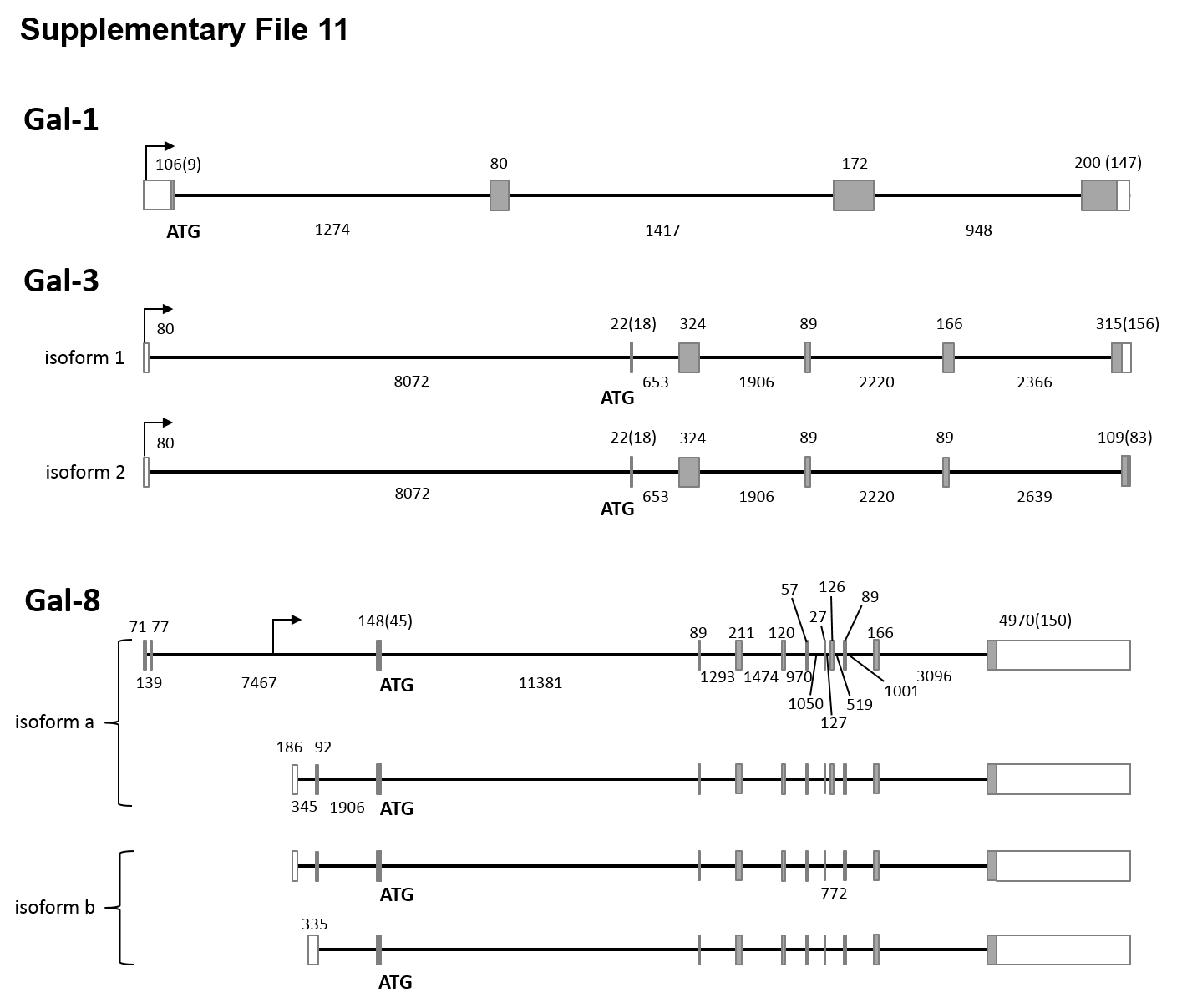

Supplement: Supplementary file 11 — Genomic organization of the genes for human Gal-1, -3 and -8. The organization of the genes of human Gal-1 (NM002305.3), Gal-3 (NM002306.3; NM001177388.1) and Gal-8 (NM201545.2; NM006499.4; NM201543.2; NM201544.2) are shown schematically. All variants are used that have a consolidated status in the NCBI gene database. Exons are given as white (non-coding sequence) or grey (coding sequence) boxes. The numbers of base pairs are entered above the respective boxes, with the size of the coding sequence in brackets if the exon contains a non-coding region. Introns are depicted as black lines, with corresponding sizes given below lines. For overview, the sizes of the exons and introns of the Gal-8 gene were only shown if there is a change in base pairs. Translation start points are marked by ATG, whereas the transcription start points (TSPs) are marked by an arrow. The TSPs were chosen based on literature data (TIFF 176 kb) [file 18_2018_2856_MOESM11_ESM.tif]

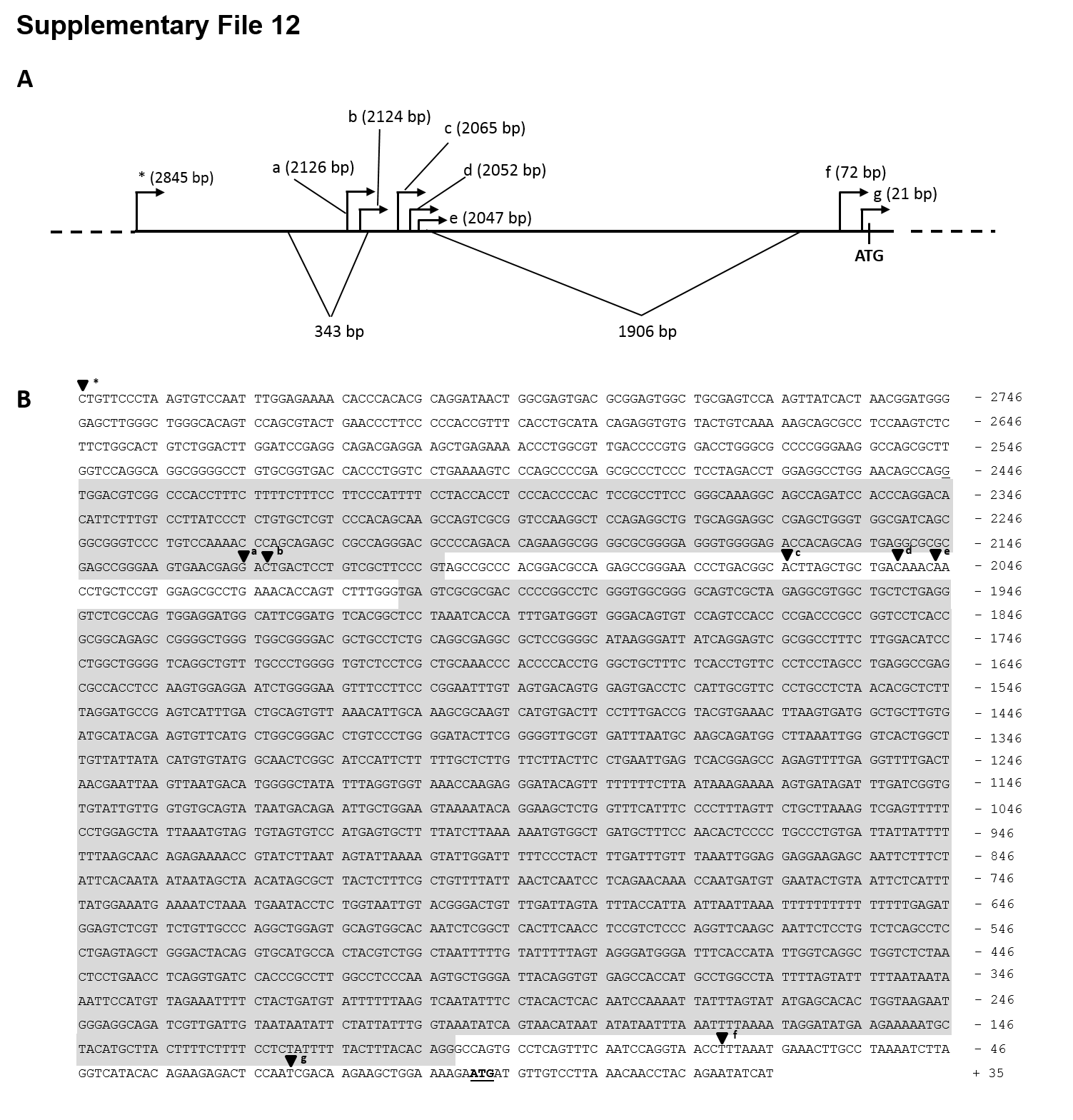

Supplement: Supplementary file 12 — Transcription start points (TSPs) of the gene for human Gal-8. Shown is the 5´ UTR region of the human Gal-8 on genomic level. The translation start point is denoted by ATG. (A) The exact position of each of the known TSP (arrows) is given in brackets as distance in basepairs relative to ATG. TSPs were given in the literature or detected experimentally via GeneRACER (Rapid amplification of cDNA ends) position c was found in the human PC-3 cell line, positions a, d, e, f, g in the human DLD-1 cell line. In both cell lines, TSP b, too, was found. Literature data report presence of two transcripts whose origin is the site labeled by (*), differing by a deletion of the indicated 343 bp-long sequence. The indicated 1906 bp-long sequence is not present in transcripts starting from the sites denoted as a-e. (B) The sequence for the 5´ UTR of human Gal-8 is given, the positions of the different TSPs (*, a-g) being marked by black triangles. The positions on the right are in relation to the translation start, i.e. ATG (bold, underlined). Grey-shaded sequences indicate areas that are not represented in transcripts (see A) (TIFF 424 kb) [file 18_2018_2856_MOESM12_ESM.tif]
