# Supplementary material for: Galectin-8 induces functional disease markers in human osteoarthritis and cooperates with galectins-1 and -3
Source: Cell Mol Life Sci. 2018 Jun 22;75(22):4187–205. doi: 10.1007/s00018-018-2856-2 (PMC6182346; doi:10.1007/s00018-018-2856-2)
Supplement: Supplementary file 8 — Comparison of the sets of glycogenes regulated by Gal-8S, Gal-3 or Gal-1 as determined by microarray analysis. The ratios between mRNA levels of glycogenes in Gal-8S-, Gal-3- or Gal-1-treated versus untreated chondrocytes as well as p-values, corrected for multiple hypothesis testing by the Benjamini–Hochberg method, are given. Data from Gal-3 and Gal-1-treated chondrocytes were reproduced from GEO (accession numbers: Gal-3: GSE85254, Gal-1: GSE68760) (PDF 74 kb) [file 18_2018_2856_MOESM8_ESM.pdf]

Supplementary File 8

N-glycosylation

Symbol	Entrez ID	Gene name	Gal-8S		Gal-3		Gal-1	
			adj. p-value	Ratio treated/untreated	adj. p-value	Ratio treated/untreated	adj. p-value	Ratio treated/untreated
ST3GAL4	6484	ST3 b-galactoside a2,3-sialyltransferase 4	6.E-05	**2.4**	1.E-02	1.8	4.E-02	1.5
FUT4	2526	fucosyltransferase 4 (alpha (1,3) fucosyltransferase, myeloid-specific)	1.E-02	**2.4**	3.E-03	2.5	-	-
B4GALT1	2683	UDP-Gal:betaGlcNAc beta 1,4- galactosyltransferase, polypeptide 1	1.E-05	**2.3**	8.E-03	2.4	-	-
B3GNT2	10678	UDP-GlcNAc:betaGal beta-1,3-N-acetylglucosaminyltransferase 2	5.E-04	**1.7**	2.E-02	1.6	-	-
FUT10	84750	fucosyltransferase 10 (a1,3-fucosyltransferase)	3.E-02	**0.7**	-	-	5.E-02	0.7
FUT8	2530	fucosyltransferase 8 (a1,6-fucosyltransferase)	3.E-02	**0.7**	-	-	1.E-02	0.4
ST6GAL1	6480	ST6 b-galactosamide a2,6-sialyltranferase 1	2.E-01	**0.5**	-	-	2.E-02	0.1
ALG13	79868	ALG13, UDP-N-acetylglucosaminyltransferase subunit	-	**-**	3.E-02	1.5	-	-
B4GALT3	8703	UDP-Gal:bGlcNAc b1,4-galactosyltransferase, polypeptide 3	-	**-**	1.E-02	1.5	4.E-03	1.9
B3GNT7	93010	UDP-GlcNAc:betaGal beta-1,3-N-acetylglucosaminyltransferase 7	-	**-**	4.E-02	1.5	-	-
B4GALT5	9334	UDP-Gal:bGlcNAc b1,4-galactosyltransferase, polypeptide 5	-	**-**	-	-	1.E-02	1.9
ALG10; ALG10B	144245; 84920	asparagine-linked glycosylation 10, a1,2-glucosyltransferase homolog (*S. pombe*); asparagine-linked glycosylation 10, a1,2-glucosyltransferase homolog B (yeast)	-	**-**	-	-	4.E-02	0.7
FUT11	170384	fucosyltransferase 11 (a1,3-fucosyltransferase)	-	**-**	-	-	5.E-02	0.6
ALG9	79796	asparagine-linked glycosylation 9, a1,2-mannosyltransferase homolog (*S. cerevisiae*)	-	**-**	-	-	3.E-02	0.4
ALG10B	144245	asparagine-linked glycosylation 10, a1,2-glucosyltransferase homolog B (yeast)	-	**-**	-	-	2.E-02	0.4
B4GALT2	8704	UDP-Gal:bGlcNAc b1,4- galactosyltransferase, polypeptide 2	-	**-**	-	-	2.E-02	0.3
ALG6	29929	asparagine-linked glycosylation 6, a1,3-glucosyltransferase homolog (*S. cerevisiae*)	-	**-**	-	-	2.E-02	0.3

O-glycosylation

Symbol	Entrez ID	Gene name	Gal-8S		Gal-3		Gal-1	
			adj. p-value	Ratio treated/untreated	adj. p-value	Ratio treated/untreated	adj. p-value	Ratio treated/untreated
ST3GAL1	6482	ST3 b-galactoside a2,3-sialyltransferase 1	2.E-06	**4.5**	1.E-03	4.1	2.E-04	4.7
POMGNT1	55624	protein O-linked mannose b1,2-N-acetylglucosaminyltransferase	4.E-04	**1.6**	4.E-03	1.7	1.E-02	1.8
B3GNT9	84752	UDP-GlcNAc:bGal b1,3-N-acetylglucosaminyltransferase 9	7.E-02	**0.7**	-	-	1.E-02	0.4
B3GLCT	145173	beta 3-glucosyltransferase	2.E-02	**0.6**	-	-	-	-
B3GAT1	27087	b1,3-glucuronyltransferase 1 (glucuronosyltransferase P)	-	**-**	-	-	4.E-02	1.8
B3GAT2	135152	b1,3-glucuronyltransferase 2 (glucuronosyltransferase S)	-	**-**	-	-	4.E-02	0.6
B3GALTL	145173	b1,3-galactosyltransferase-like	-	**-**	-	-	3.E-02	0.4
B3GNT1	11041	UDP-GlcNAc:bGal b1,3-N-acetylglucosaminyltransferase 1	-	**-**	-	-	2.E-02	0.3

Glycolipids

Symbol	Entrez ID	Gene name	Gal-8S		Gal-3		Gal-1	
			adj. p-value	Ratio treated/untreated	adj. p-value	Ratio treated/untreated	adj. p-value	Ratio treated/untreated
A4GALT	53947	a1,4-galactosyltransferase	8.E-05	**2.6**	3.E-03	1.9	2.E-02	2.0
B3GNT5	84002	UDP-GlcNAc:bGal b1,3-N-acetylglucosaminyltransferase 5	2.E-02	**2.1**	-	-	2.E-02	2.8
ST6GALNAC6	30815	ST6 (a-N-acetyl-neuraminyl-2,3-b-galactosyl-1,3)-N-acetylgalactosaminide a-2,6-sialyltransferase 6	4.E-04	**0.6**	-	-	1.E-02	0.6
ST6GALNAC5	81849	ST6 (a-N-acetyl-neuraminyl-2,3-b-galactosyl-1,3)-N-acetylgalactosaminide a-2,6-sialyltransferase 5	1E-03	**0.5**	-	-	3.E-02	0.7
GBGT1	26301	globoside a1,3-N-acetylgalactosaminyltransferase 1	4.E-06	**0.5**	-	-	2.E-02	0.7
B3GALNT1	8706	b1,3-N-acetylgalactosaminyltransferase 1 (globoside blood group)	6.E-02	**0.4**	-	-	1.E-02	0.1
ST3GAL5	8869	ST3 beta-galactoside alpha-2,3-sialyltransferase 5	7.E-03	**0.4**	2.E-02	0.5	-	-

Mannosidases

Symbol	Entrez ID	Gene name	Gal-8S		Gal-3		Gal-1	
			adj. p-value	Ratio treated/untreated	adj. p-value	Ratio treated/untreated	adj. p-value	Ratio treated/untreated
MAN1A1	4121	mannosidase, alpha, class 1A, member 1	3.E-05	**3.5**	2.E-03	2.6	-	-
MAN2B2	23324	a-mannosidase, class 2B, member 2	8.E-02	**0.7**	-	-	8.E-03	0.4
MAN2A2	4122	a-mannosidase, class 2A, member 2	2.E-02	**0.7**	-	-	9.E-03	0.4
MAN2A1	4124	mannosidase alpha class 2A member 1	1.E-03	**0.6**	-	-	-	-
MANBA	4126	a-mannosidase, b A, lysosomal	1.E-02	**0.5**	-	-	1.E-02	0.4
MAN1B1	11253	a-mannosidase, class 1B, member 1	3.E-03	**0.5**	-	-	1.E-02	0.4
MANSC1	54682	MANSC domain containing 1	9.E-04	**0.4**	-	-	2.E-02	0.2
MAN1C1	57134	a-mannosidase, class 1C, member 1	2.E-04	**0.2**	-	-	3.E-04	0.2
MAN1A2	10905	a-mannosidase, class 1A, member 2	-	**-**	-	-	2.E-02	0.5

Sialic acid (donor) synthesis/processing

Symbol	Entrez ID	Gene name	Gal-8S		Gal-3		Gal-1	
			adj. p-value	Ratio treated/untreated	adj. p-value	Ratio treated/untreated	adj. p-value	Ratio treated/untreated
SIAE	54414	sialic acid acetylesterase	9.E-02	**0.69**	-	-	1.E-02	0.4
NANS	54187	N-acetylneuraminic acid synthase	-	**-**	-	-	4.E-02	1.8
CMAS	55907	cytidine monophosphate N-acetylneuraminic acid synthetase	-	**-**	-	-	2.E-02	0.6

Lectins

Symbol	Entrez ID	Gene name	Gal-8S		Gal-3		Gal-1	
			adj. p-value	Ratio treated/untreated	adj. p-value	Ratio treated/untreated	adj. p-value	Ratio treated/untreated
SELE	6401	E-selectin (CD62E)	6E-06	**28.7**	2.E-03	74.3	3.E-07	323.3
CLEC2D	29121	C-type lectin domain family 2, member D	4.E-02	**2.4**	3.E-02	1.5	-	-
LGALS8	3964	lectin, galactoside-binding, soluble, 8	2.E-02	**1.9**	1.E-02	1.8	-	-
LGALS3BP	3959	galectin 3 binding protein	9.E-04	**1.8**	-	-	-	-
LGALSL	29094	galectin like	2.E-01	**1.6**	-	-	-	-
CLEC18B	497190	C-type lectin domain family 18 member B	5.E-02	**0.7**	-	-	-	-
CLEC18C	283971	C-type lectin domain family 18 member C	5.E-02	**0.7**	-	-	-	-
CLEC18A	348174	C-type lectin domain family 18 member A	5.E-02	**0.7**	-	-	-	-
SELP	6403	P-selectin (granule membrane protein 140kDa, antigen CD62P)	1.E-01	**0.7**	-	-	1.E-02	0.4
COLEC12	81035	collectin sub-family member 12	1.E-05	**0.2**	-	-	2.E-03	0.2
CLEC3A	10143	C-type lectin domain family 3, member A	1.E-06	**0.1**	-	-	9.E-04	0.2
LMAN1	3998	lectin, mannose-binding, 1	-	**-**	2.E-03	1.7	-	-
OLR1	4973	oxidized low density lipoprotein (lectin-like) receptor 1	-	**-**	-	-	2.E-02	5.5
HSPC159	29094	galectin-related protein (GRP)	-	**-**	-	-	2.E-02	3.2
KLRD1	3824	killer cell lectin-like receptor subfamily D, member 1	-	**-**	-	-	1.E-02	2.1
CLEC4E	26253	C-type lectin domain family 4, member E	-	**-**	-	-	7.E-03	1.9
KLRK1	22914	killer cell lectin-like receptor subfamily K, member 1	-	**-**	-	-	5.E-02	1.6
LGALS3	3958	lectin, galactoside-binding, soluble, 3 (galectin-3)	-	**-**	-	-	3.E-02	1.5
KLRC1	3821	killer cell lectin-like receptor subfamily C, member 1	-	**-**	-	-	4.E-02	1.5
KLRG1	10219	killer cell lectin-like receptor subfamily G, member 1	-	**-**	-	-	3.E-02	0.7
MLEC	9761	malectin	-	**-**	-	-	3.E-02	0.4
CLEC3B	7123	C-type lectin domain family 3, member B	-	**-**	-	-	2.E-03	0.3
