# Supplementary material for: Galectin-8 induces functional disease markers in human osteoarthritis and cooperates with galectins-1 and -3
Source: Cell Mol Life Sci. 2018 Jun 22;75(22):4187–205. doi: 10.1007/s00018-018-2856-2 (PMC6182346; doi:10.1007/s00018-018-2856-2)
Supplement: Supplementary file 9 — Comparison of the sets of genes relevant for degradative and inflammatory processes regulated by Gal-8S, Gal-3, or Gal-1 as determined by microarray analysis. The ratios between mRNA levels of OA relevant genes in Gal-8S-, Gal-3- or Gal-1-treated versus untreated chondrocytes as well as p-values, corrected for multiple hypothesis testing by the Benjamini–Hochberg method, are given. Data from Gal-3 and Gal-1-treated chondrocytes were reproduced from GEO (accession numbers: Gal-3: GSE85254, Gal-1: GSE68760) (PDF 67 kb) [file 18_2018_2856_MOESM9_ESM.pdf]

Supplementary File 9

Cartilage- and OA-related genes

Symbol	Entrez ID	Gene name	Gal-8S adj. p-value	Gal-8S Ratio treated/untreated	Gal-3 adj. p-value	Gal-3 Ratio treated/untreated	Gal-1 adj. p-value	Gal-1 Ratio treated/untreated
COL7A1	1294	collagen, type VII, alpha 1	5.E-03	**2.4**	1.E-03	3.7	1.E-03	4.7
COL22A1	169044	collagen, type XXII, alpha 1	2.E-02	**1.5**	-	-	6.E-03	1.8
PCOLCE2	26577	procollagen C-endopeptidase enhancer 2	1.E-01	**0.7**	-	-	2.E-02	0.4
COL6A3	1293	collagen, type VI, alpha 3	3.E-01	**0.7**	-	-	2.E-02	0.2
COL5A1	1289	collagen, type V, alpha 1	1.E-01	**0.7**	-	-	1.E-02	0.3
COL6A1	1291	collagen, type VI, alpha 1	1.E-01	**0.7**	-	-	1.E-02	0.4
COL1A2	1278	collagen, type I, alpha 2	3.E-01	**0.7**	-	-	8.E-03	0.2
PCOLCE	5118	procollagen C-endopeptidase enhancer	3.E-02	**0.6**	-	-	3.E-03	0.5
COL12A1	1303	collagen, type XII, alpha 1	4.E-02	**0.6**	-	-	2.E-03	0.2
COL8A2	1296	collagen, type VIII, alpha 2	6.E-02	**0.6**	-	-	-	-
COL9A1	1297	collagen, type IX, alpha 1	2.E-01	**0.6**	-	-	-	-
COL21A1	81578	collagen, type XXI, alpha 1	9.E-04	**0.6**	-	-	2.E-02	0.5
COL9A3	1299	collagen type IX alpha 3	5.E-02	**0.5**	-	-	-	-
COL5A2	1290	collagen, type V, alpha 2	2.E-02	**0.4**	-	-	2.E-02	0.3
COL10A1	1300	collagen, type X, alpha 1	5.E-02	**0.3**	-	-	2.E-02	0.2
COL14A1	7373	collagen, type XIV, alpha 1	5.E-03	**0.3**	-	-	1.E-02	0.2
COL11A1	1301	collagen, type XI, alpha 1	2.E-03	**0.2**	-	-	8.E-03	0.1
COL5A3	50509	collagen, type V, alpha 3	-	**-**	-	-	5.E-02	1.9
COL1A1	1277	collagen, type I, alpha 1	-	**-**	-	-	1.E-02	0.5
COL8A1	1295	collagen, type VIII, alpha 1	-	**-**	-	-	1.E-02	0.4
COL15A1	1306	collagen, type XV, alpha 1	-	**-**	-	-	2.E-02	0.2
COL8A2	1296	collagen, type VIII, alpha 2	-	**-**	-	-	3.E-03	0.2
ACAN	176	aggrecan	8.E-05	**0.3**	-	-	5.E-02	0.5
LAMB3	3914	laminin, beta 3	3.E-08	**29.8**	2.E-04	19.0	3.E-04	23.6
LAMC2	3918	laminin, gamma 2	9.E-02	**1.9**	3.E-02	1.8	3.E-02	3.5
LAMC1	3915	laminin, gamma 1 (formerly LAMB2)	2.E-02	**0.7**	-	-	2.E-02	0.5
LAMB2	3913	laminin, beta 2 (laminin S)	1.E-02	**0.6**	-	-	3.E-02	0.6
LAMA4	3910	laminin, alpha 4	9.E-04	**0.6**	-	-	5.E-03	0.4
LAMB1	3912	laminin, beta 1	2.E-03	**0.3**	-	-	5.E-03	0.2
LAMA3	3909	laminin, alpha 3	-	**-**	-	-	3.E-02	2.4
FSD1L	83856	fibronectin type III and SPRY domain containing 1-like	1.E-02	**2.9**	3.E-02	1.5	6.E-03	8.9
FNDC3B	64778	fibronectin type III domain containing 3B	3.E-07	**2.6**	1.E-03	2.8	6.E-04	2.8
FNDC4	64838	fibronectin type III domain containing 4	3.E-05	**0.5**				
DCN	1634	decorin	8.E-02	**0.5**	-	-	2.E-02	0.2
MMP10	4319	matrix metallopeptidase 10 (stromelysin 2)	3.E-06	**12.5**	1.E-03	8.0	5.E-04	10.6
MMP12	4321	matrix metallopeptidase 12 (macrophage elastase)	3.E-07	**12.4**	7.E-04	8.3	7.E-05	15.8
MMP13	4322	matrix metallopeptidase 13 (collagenase 3)	2.E-04	**8.3**	2.E-04	4.7	-	-
MMP1	4312	matrix metallopeptidase 1 (interstitial collagenase)	6.E-04	**7.5**	5.E-03	5.0	-	-
MMP7	4316	matrix metallopeptidase 7 (matrilysin, uterine)	7.E-03	**2.7**	3.E-02	2.6	5.E-02	1.4
MMP16	4325	matrix metallopeptidase 16	7.E-04	**0.7**	-	-	-	-
MMP11	4320	matrix metallopeptidase 11	3.E-04	**0.6**	-	-	-	-
MMP9	4318	matrix metallopeptidase 9	-	**-**	-	-	3.E-02	3.7
MMP19	4327	matrix metallopeptidase 19	-	**-**	-		3.E-02	2.4
MMP25	64386	matrix metallopeptidase 25	-	**-**	-	-	4.E-02	2.1
ADAMTS9	56999	ADAM metallopeptidase with thrombospondin type 1 motif 9	8.E-07	**4.6**	3.E-03	4.5	-	-
ADAMTS4	9507	ADAM metallopeptidase with thrombospondin type 1 motif, 4	5.E-02	**2.4**	4.E-02	3.2	4.E-03	6.4
ADAMTS12	81792	ADAM metallopeptidase with thrombospondin type 1 motif 12	2.E-02	**0.7**	-	-	-	-
ADAMTS13	11093	ADAM metallopeptidase with thrombospondin type 1 motif 13	2.E-03	**0.7**	-	-	-	-
ADAMTS1	9510	ADAM metallopeptidase with thrombospondin type 1 motif 1	2.E-01	**0.5**	-	-	-	-
ADAMTS5	11096	ADAM metallopeptidase with thrombospondin type 1 motif, 5	-	**-**	-	-	1.E-02	0.2
BMP2	650	bone morphogenetic protein 2	6.E-04	**3.2**	2.E-04	6.0	2.E-03	5.7
BMP6	654	bone morphogenetic protein 6	7.E-03	**3.2**	2.E-02	2.9	2.E-03	4.3
BMPR1B	658	bone morphogenetic protein receptor, type IB	6.E-04	**3.0**	-	-	4.E-04	3.4
BMP1	649	bone morphogenetic protein 1	3.E-03	**1.5**	-	-	8.E-03	1.8
BMPR1A	657	bone morphogenetic protein receptor, type IA	2.E-01	**0.7**	-	-	2.E-02	0.2
BMPR2	659	bone morphogenetic protein receptor type 2	1.E-02	**0.6**	-	-	-	-
BMP4	652	bone morphogenetic protein 4	4.E-02	**0.5**	-	-	5.E-03	0.2
BMP5	653	bone morphogenetic protein 5	-	**-**	2.E-02	1.5	1.E-02	2.9
BMP8A	353500	bone morphogenetic protein 8a	-	**-**	-	-	2.E-02	1.7
BMP7	655	bone morphogenetic protein 7	-	**-**	-	-	2.E-02	1.6
TGFBR2	7048	transforming growth factor beta receptor 2	2.E-04	**0.6**	-	-	-	-
TGFBR3	7049	transforming growth factor beta receptor 3	2.E-03	**0.5**	2.E-03	0.5	-	-
TGFB3	7043	transforming growth factor, beta 3	1.E-05	**0.3**	-	-	3.E-02	0.5
TGFB2	7042	transforming growth factor, beta 2	1.E-05	**0.3**	-	-	1.E-02	0.5
CTGF	1490	connective tissue growth factor	2.E-05	**0.1**	9.E-03	0.3	-	-
TGFA	7039	transforming growth factor, alpha	-	**-**	-	-	3.E-02	1.6
IGF2BP2	10644	insulin like growth factor 2 mRNA binding protein 2	2.E-02	**0.7**	-	-	-	-
IGF2;<br>INS-IGF2	3481;<br>723961	insulin-like growth factor 2 (somatomedin A);<br>INS-IGF2 readthrough transcript	1.E-01	**0.5**	-	-	4.E-02	0.3
IGFBP7	3490	insulin-like growth factor binding protein 7	-	**-**	3.E-02	2.1	-	-
IGFBP1	3484	insulin-like growth factor binding protein 1	-	**-**	5.E-03	1.9	-	-
IGFBP6	3489	insulin-like growth factor binding protein 6	-	**-**	-	-	3.E-02	0.6
IGF1R	3480	insulin-like growth factor 1 receptor	-	**-**	-	-	3.E-02	0.4
IGFBP5	3488	insulin-like growth factor binding protein 5	-	**-**	-	-	1.E-02	0.3
IGF1	3479	insulin-like growth factor 1 (somatomedin C)	-	**-**	1.E-01	1.3	-	-
IGF2BP1	10642	insulin-like growth factor 2 mRNA binding protein 1	-	**-**	1.E-01	1.2	-	-
IGF2BP3	10643	insulin-like growth factor 2 mRNA binding protein 3	-	**-**	3.E-01	1.1	-	-
LECT1	11061	chondromodulin	8.E-02	**0.5**	-	-	-	-
TNF	7124	tumor necrosis factor	3.E-01	**1.8**	-	-	7.E-03	23.6
TLR2	7097	toll-like receptor 2	9.E-06	**26.5**	3.E-04	20.3	3.E-02	5.9
TLR1	7096	toll-like receptor 1	4.E-02	**1.7**	8.E-03	2.1	-	-
TLR5	7100	toll-like receptor 5	1.E-02	**0.6**	-	-	-	-
TLR4	7099	toll-like receptor 4	2.E-03	**0.4**	-	-	-	-
TLR3	7098	toll-like receptor 3	-	**-**	3.E-03	2.0	-	-
ITGB8	3696	integrin, beta 8	2.E-02	**1.8**	3.E-03	1.8	-	-
ITGB3	3690	integrin, beta 3 (platelet glycoprotein IIIa, antigen CD61)	2.E-03	**1.7**	2.E-02	1.6		-
CIB2	10518	calcium and integrin binding family member 2	1.E-07	**1.5**	-	-	2.E-02	0.6
ITGB3BP	23421	integrin beta 3 binding protein (beta3-endonexin)	1.E-01	**0.7**	-	-	8.E-03	0.3
ITGA2	3673	integrin subunit alpha 2	2.E-02	**0.6**	-	-	-	-
ITGAE	3682	integrin, alpha E (antigen CD103, human mucosal lymphocyte antigen 1; alpha	3.E-02	**0.6**	-	-	2.E-02	0.4
ITGA3	3675	integrin, alpha 3 (antigen CD49C, alpha 3 subunit of VLA-3 receptor)	5.E-02	**0.6**	-	-	9.E-03	0.5
ITGB5	3693	integrin subunit beta 5	2.E-03	**0.5**	-	-	-	-
ITGA6	3655	integrin subunit alpha 6	1.E-02	**0.4**	-	-	-	-
ITGA11	22801	integrin, alpha 11	8.E-04	**0.4**	-	-	2.E-02	0.5
ITGA10	8515	integrin, alpha 10	6.E-05	**0.2**	-	-	1.E-04	0.1
ITGBL1	9358	integrin, beta-like 1 (with EGF-like repeat domains)	5.E-04	**0.2**	-	-	1.E-03	0.1
ITGA5	3678	integrin, alpha 5 (fibronectin receptor, alpha polypeptide)	-	**-**	1.E-02	1.7	1.E-02	1.8
ITGA1	3672	integrin, alpha 1	-	**-**	1.E-02	1.5	1.E-02	2.0
ITGAM	3684	integrin, alpha M (complement component 3 receptor 3 subunit)	-	**-**	-	-	1.E-02	8.4
ITGB1	3688	integrin, beta 1 (fibronectin receptor, beta polypeptide, antigen CD29 includes MDF2, MSK12)	-	**-**	-	-	5.E-02	0.5
ITGB5	3693	integrin, beta 5	-	**-**	-	-	5.E-03	0.4
ITFG1	81533	integrin alpha FG-GAP repeat containing 1	-	**-**	-	-	4.E-02	0.4
ITGB1BP1	9270	integrin beta 1 binding protein 1	-	**-**	-	-	3.E-02	0.4
