# Supplementary material for: Galectin-8 induces functional disease markers in human osteoarthritis and cooperates with galectins-1 and -3
Source: Cell Mol Life Sci. 2018 Jun 22;75(22):4187–205. doi: 10.1007/s00018-018-2856-2 (PMC6182346; doi:10.1007/s00018-018-2856-2)
Supplement: Supplementary file 14 — Compilation of computationally detected putative binding sites for transcription factors (TFs) in the promoter and intron regions of the genes for human Gal-1, -3 and -8. The analyzed regions were -2000 to +97 for Gal-1, -2500 for Gal-3 and -2500 for Gal-8 (promoter regions) and the sequences of the introns of the genes of Gal-1 (3 introns), -3 (5 introns) and -8 (9 introns). A list of all detected TFs for either Gal-1 or Gal-3 was published previously [17, 18]. The putative binding sites were compared regarding to their shared or specific occurrence in the promoter and intron regions of the galectins (PDF 105 kb) [file 18_2018_2856_MOESM14_ESM.pdf]

Supplementary File 14: Compilation of computationally detected putative binding sites for transcription factors (TFs) in the promoter and intron regions of the genes for human Gal-1, -3 and -8. The analyzed regions were -2000 to +97 for Gal-1, -2500 for Gal-3 and -2500 for Gal-8 (promoter regions) and the sequences of the introns of the genes of Gal-1 (3 introns), -3 (5 introns) and -8 (9 introns). A list of all detected TFs for either Gal-1 or Gal-3 was published previously (Toegel et al., 2016; Weinmann et al., 2016). The putative binding sites were compared regarding to their shared or specific occurrence in the promoter and intron regions of the galectins.

	Putative binding sites common for Gal-1, -3 and -8
promoter	ACAAT, AP1, BARBIE, BCL6, BRIGHT, BRN2, BRN3, BRN5, BSX, CDP, CHOP, CREB2, CREL, CRX, CSRNP1, CTCF, DLX1, DLX3, DREAM, E2F, E2F1, E2F4, EKLF, EN1, FAST1, GATA1, GATA3, GC, GCM1, GFI1, GSH1, GSH2, HHEX, HIC1, HMGIY, HMX3, HNF1, HOXB9, HOXC13, HOXC4, HOXC9, HSF2, IK3, IR2_NGRE, IRF2, ISL2, JUNB, KKLF, KLF7, LEF1, LHX3, LRRFIP1, MEF2, MEL1, MSX, MTBF, MYBL1, MZF1, NANOG, NF1, NFAT, NFAT5, NFY, NKX12, NKX25, NKX31, NKX61, NMP4, OLF1, P53, PAX3, PAX6, PCE1, PHOX2, PPARG, PRDM1, PSE, PURα, S8, SMARCA3, SOX3, SP1, SPI1, SREBP, SRF, STAT, TCFAP2A, VMYB, XBOX, YB1, ZBED4, ZBP89, ZBTB3, ZF5, ZFP410, ZNF282, ZNF300
(introns)	(AARE, AHRARNT, AIRE, AML1, AML2, AP1, AP2, AP4, ARID5A, ARNTL, ATBF1, ATF, ATOH1, BARHL1, BCL6, BKLF, BLIMP1, BNC, BRIGHT, BRN2, BRN3, BRN4, BRN5, BSX, BTEB3, CAAT, CARF, CART1, CDP, CDX1, CDX2, CEBP, CEBPE_ATF4, CHOP, CHR, CLEAR, CMYB, COUP, CP2, CREB, CREL, CRX, CSRNP1, CTCF, DBP, DEC1, DEC2, δEF1, DLX2, DLX3, DLX4, DMP1, DMRT3, DREAM, DUX4, E2F, E2F3, E2F4, E2F7, E4F, EBF1, EGR1, EGR2, EKLF, ELK1, ER, ESRRA, ESRRB, ETV1, EVI1,, EVX1, FAST1, FHXB, FOSL1, FOXO1, FOXP1_ES, FREAC7, FTF, GABP, GATA1, GATA3, GBX1, GC, GCM1, GFI1, GKLF, GLIS2, GLIS3, GRHL1, GRHL2, GRHL3, HAS, HBP1, HDBP1_2, HEN1, HIC1, HMBOX, HMGIY, HMX2, HMX3, HNF1, HNF3, HNF6, HOMEZ, HOX1-3, HOXA3, HOXB3, HOXB4, HOXB5, HOXC8, HOXC9, HOXC13, HOXD10, HSF1, HSF2, IK1, IK2, IK3, INSM1, IPF1, IR1_NGRE, IRF2, IRF4, ISL1, ISL2, JARID2, JUNB, KKLF, KLF2, KLF6, KLF7, KLF12, LACTOFERRIN, LBX2, LEF1, LHX2, LHX4, LHX9, LRRFIP1, LTSM, LYF1, LYL1_E12, MARE, MAZ, MAZR, MEIS1, MEL1, MESP1_2, MIT, MIXL1, MIZ1, MIZF, MOK2, MSX2, MSX3, MTBF, MYBL1, MYOD, MYOGENIN, MYRF, MYT1, MZF1, NACA1, NANOG, NBRE, NEUROG, NF1, NFAT5, NFkB, NFkB50, NFY, NGN_NEUROD, NKX11, NKX12, NKX25, NKX26, NKX31, NKX61, NKX63, NM23, NMP4, NOBOX, NR2F6, NRL, NRSF, NXF_ARNT, OC2, OCT1, OCT3_4, OLF1, OLIG2, OVOL1, P53, PARAXIS, PAX3, PAX4, PAX4_PD, PAX5, PAX6, PAX6_HD, PBX1_MEIS1, PBX3, PCE1, PDEF, PEA3, PHOX2, PIT1, PLAG1, PLAGL1, PLZF, PNR, POU2F3, POU6F2, PPARG, PRDM1, PRDM14, PROP1, PURα, RAR_RXR, RAX, RBPJK, REV-ERBA, RFX4, RORA, RP58, RREB1, S8, SALL1, SALL2, SATB1, SF1, SIX2, SIX3, SMAD3, SMAD4, SMARCA3, SOX1, SOX5, SOX7, SOX9, SOX15, SP1, SP2, SP4, SPI1, SPZ1, SREBP, SRF, SRY, STAT3, STAT5A, STAT5B, SZF1, TAL1_E2A, TAL1αE47, TCF2, TCFAP2A, TCFAP2B, TCFCP2L1, TCFE2A, TEF, TEF1, TH1E47, THRB, TLX1, TST1, VAX2, VBP, VDR_RXR, VERBA, VMYB, WHN, WT1, XBOX, XFD2, YB1, YY1, YY2, ZBED4, ZBP89, ZBTB3, ZBTB7, ZF5, ZFP410, ZFP57, ZFP652, ZFX, ZIC2, ZIC2, ZIC3, ZKSCAN3, ZNF217, ZNF219, ZNF263, ZNF282, ZNF300, ZNF35, ZNF354C, ZSCAN10)

	Putative binding sites common for Gal-1 and Gal-3
promoter	AHRARNT, ARE, ATBF1, ATF6, BKLF, CDX1, CHR, CP2, δEF1, E2F3, EBF1, EGR1, FREAC7, GKLF, HDBP1_2, HFH1, HOXA5, HOXB8, HSF1, INSM1, KAISO, KLF6, LHX1, LHX2, LTSM, MAFA, MAZR, MESP1_2, MIZ1, MYOD, NBRE, NFκB65, NKX11, PIT1, PLAG1, PLAGL1, PREB, PROP1, SMAD3, SPZ1, STAT3, STAT5A, STAT6, TCF7L1, TLX1, VDR_RXR, ZBTB7, ZFX
(introns)	(CAR_RXR, CEBPB, CREB3L2, EGR3, GSH1, GSH2, HAND2_E12, HESX1, NFATC1, NKX29, NRF1, OTX2, PDX1, PTF1, SOX10, ZBED1, ZIC3)
	Putative binding sites common for Gal-1 and Gal-8
promoter	AARE, AP4, AREB6, CJUN_ATF2, CREB1, DLX5, E47, FOXP1, GLI3, GRE, GSC, HDGF, ISGF3G, KLF12, LBX2, MAZ, MEIS1, MIT, MOK2, MSX2, MYT1L, NACA1, NFκB, PAX4, PBX1, PLU1_JARID1B, PNR, RAX, RREB1, SOX5, SP4, SPT, TGIF, THR, VERBA, WT1, XBP1, YY1, ZKSCAN3, ZNF263
(introns)	(ACAAT, ARE, BACH2, CHREBP_MLX, CJUN_ATF2, CLOX, ESR2, ETS2, GABPA, GATA2, GLI3, GMEB2, HDGF, HES1, HFH3, HOXA5, HOXB7, HOXD1, LMX1B, MASH1, MEIS1A_HOXA9, NEUROD1, NFκB65, NUDR, PPAR_RXR, PRRX1, RFX1, RFX3, SCRT2, SOX2, TAXCREB, TCF12, TCF21, TCFAP2C)
	Putative binding sites common for Gal-3 and Gal-8
promoter	BACH2, BATF, BHLHA15, CDX2, CEBPB, CPHX, DBP, DMP1, E4BP4, ERG, ESRRA, ETV1, EVI1, FAC1, FTF, GATA2, GBX1, GFI1B, GRHL2, HBP1, HLF, HMGA, HMX2, HNF3B, HNF4G, HNF6, HOXA3, HOXA9, HOXB7, HOXC8, HOXC9, IRF3, JARID2, MASH1, MEIS1, MEOX1, MSX1, MYT1, NKX63, NOBOX, OC2, OCT1, OCT3_4, OVOL1, PARAXIS, PAX7, PHOX2A, PLZF, POU3F3, REX1, RFX1, RFX4, RFX5, SATB1, SCX, SF1, SIX1, SOX6, SOX9, SOX21, SPIB, SRY, TEAD4, TEF TST1, VBP, ZFP652, ZNF217, ZTRE
(introns)	(ALX3, ALX4, AML3, AREB6, ATF6, BAPX1, BARBIE, BARX1, BARX2, BATF, BHLHA15, CABL, CDE, CEBPA, CEBPD, CEBPE, CEBPG, CKROX, CMYC, CPHX, CREB2, DLX1, DLX5, DMRT1, DMRT2, DMRT5, DMRT7, E47, E4BP4, ELF5, EMX2, EN1, EN2, EOMES, ERG, ERR, ESX1, ETV4, EVX2, FAC1, FOXA1, FOXJ1, FOXP1, FOXP2, FREAC2, FREAC3, GATA4, GBX2, GC_SBE, GFI1B, GLI1, GRE, HELT, HFH1, HHEX, HIF1, HLF, HMGA, HNF4, HNF4A, HOX_PBX, HOXA4,, HOXA9, HOXA10, HOXA13, HOXB4, HOXB6, HOXB8, HOXB9, HOXC4, HOXC6, HOXC10, HOXD3, HOXD8, HOXD13, HRE, IRF1, IRF3, IRF7, IRX3, IRX5, IRX6, ISGF3G, ISRE, ISX, KAISO, LHX1, LHX3, LHX6, LMX1A, MAFA, MAFB, MAFF, MAFK, MEF2, MEF3, MEIS1, MEOX1, MGA, MNT, MRF2, MSX, MTF-1, MYCMAX, MYF5, MYF6, NFAT, NFE2, NKX32, NMYC, OSNT, OSR1, OTX1, PAX2, PAX7, PAX8, PEG3, PEGASUS, PLU1_JARID1B, PRE, PREB, RARG, REX1, RFX5, RHOX6, RSRFC4, RTR, RU49, SCX, SIX1, SIX3, SL1, SOX3, SOX4, SOX6, SOX21, SOX30, SPIB, SPIC, SPT, STAF, STAT1, STAT5, STAT6, TAL1βE47, TBOX, TBX20, TCF11, TCF11MAFG, TCF7, TCF7L1, TCFAP2E, TEAD, TEF_HLF, TGIF, THAP1, THR, THRA, TIEG, TLX2, TP63, USF, VAX1, VMAF, XBP1, XFD1, XVENT2, ZBTB7, ZID, ZTRE)

	Putative binding sites specific for Gal-1
promoter	AML1, AP2, ARNTL, BTEB3, CART1, CHREBP_MLX, CKROX, CTCFL, DMRT1, EGR2, EGR3, ELK1, ER, ETV4, GABPA, GC_SBE, GLIS2, GZF1, HAS, HFH3, HIF1, HNF4, HOMEZ, HOXD13, KLF2, LACTOFERRIN, LHX4, LHX8, MEF3, MIZF, MTF-1, MYF5, MYOGENIN, NEUROD1, NKX29, NM23, NRF1, NRL, NXF_ARNT, OLIG2, PAX2, PEG3, PRDM5, PXR_RXR, RARA, RU49, RXR_RXR, SALL1,, SIX2, SOX10, SZF1, TAL1_E2A, TCFAP2E, WHN, ZIC2, ZIC3, ZNF219, ZNF354C
(introns)	(BRACH, ELF2, ESRRG, FLI, GLIS1, GTF3R4, HLXB9, HPF1, MEF3, MIF1, MRG1, PAX9, PPARA, PPARG, PRDM4, PRDM5, PRRX2, PSE, RARA, RXR_RXR, ZEC, ZNF143, ZNF202, ZNF76_143)
	Putative binding sites specific for Gal-3
promoter	AIRE, AML3, BARHL2, BARX1, BARX2, BRN4, CAAT, CDPCR3HD, CEBPE_ATF4, DICE, DLX2, DMRT3, DUX4, E2F7, EMX2, ESR2, ETS2, FHXB, FOSL2, FREAC2, GAGA, GATA4, GLIS3, GRHL3, HOXA10, HOXB3, HOXB4, HOXB13, HOXC10, HOXD8, HOXD10, IK1, IK2, IPF1, ISRE, ISX, LHX5, LHX9, MIXL1, MSX3, MYCMAX, NUDR, OSR1, PAX6_HD, PAX8, PEA3, POU6F2, PTX1, RARG, RBPJK, RORA, RP58, SL1, SOX1, SOX17, SOX4, SOX8, STAT1, TAL1βHEB, TCF11, TCF12, TCFCP2L1, TEF_HLF, THRA, TIEG, VSX1
(introns)	(BATF3, DMBX1, DMRT4, E2F2, E2F6, ETS1, HES7, HMX1, HNF4G, HOXA1, HOXA2, IRX2, JUNDM2, MEF2C, MEIS1B_HOXA9, MYBL2, NRF2, PBX1, PKNOX2, PROX1, PTX1, PXR_RXR, SIP1, SOX18, TWIST)
	Putative binding sites specific for Gal-8
promoter	ARID5A, ASCL2, BHLHB2, BLIMP1, CABL, CEBPE, CETS1P54, CLOX, CMYB, COUP, DMRT2, E2F2, ELF2, EN2, EOMES, FHXA, FOXJ1, FOXP1_ES, GRHL1, HEN1, HNF3, HNF4A, HOX_PBX, HOX1-3, HOXA2, HOXA9, HOXC5, HOXD3, IRF4, IRF7, ISL1, LMX1B, LYF1, MAFB, MAFK, MEF3, NFE2, OSNT, OTX2, PBX1_MEIS1, POU2F3, PRDM14, PRE, RAR_RXR, REV-ERBA, RFX2, RFX3, RXRA, SHOX2, SOX12, STAF, TCF11MAFG, TCFE2A, TEAD, TGIF2LX, USF1, XFD3, ZBTB7, ZEB1, ZNF35
(introns)	(AHR, ARNT, ASCL1, ASCL2, BACH1, BHLHB2, CDPCR3HD, CETS1P54, COUPTFII, CUX2, DICE, E2F1, ELF1, ETV3, FKHRL1, FOSL2, GAGA, GATA5, GSC, HFH2, HFH8, HOXA6, HOXC12, ILF1, LHX5, LHX8, MAX, MSX1, MYT1L, NFIB, NGFIC, NKX23, OBOX5, OSR2, OTP, PAX2, PBX_HOXA9, RFX2, RXRA, SHOX2, SIX4, SOX8, SOX12, SOX13, SOX14, SPDEF, TAL1βITF2, TBX5, TBX6, TEAD4, TGIF2LX, TR2, TR2_TR4, TR4, VSX1, XFD3, ZBRK1, ZEB1)
